# Dependence of the Helmholtz layer capacitance on interfacial charge density at polarisable liquid|liquid interfaces

**DOI:** 10.1039/d6sc03450k

**Published:** 2026-09-24

**Authors:** Juliana V. Díaz-Reyes, Nicolás Sanabria Rojas, Micheál D. Scanlon, Marco F. Suárez-Herrera

**Affiliations:** a Department of Chemical Sciences and Bernal Institute, University of Limerick (UL) Limerick V94 T9PX Ireland micheal.scanlon@ul.ie; b Departamento de Química, Facultad de Ciencias, Universidad Nacional de Colombia KR 30 No. 45-03, Edificio 451 Bogotá Colombia mfsuarezh@unal.edu.co

## Abstract

The physical structure of a polarisable interface between two immiscible electrolyte solutions (ITIES) is modelled using an extended Gouy–Chapman–Stern framework that incorporates asymmetric ion charges. The interface is treated as three capacitors in series: two Poisson–Boltzmann diffuse layers on each side of the aqueous|organic boundary and a Helmholtz layer that includes the dielectric saturation of solvent molecules under an intense local electric field, which reduces the Helmholtz capacitance away from the potential of zero charge (PZC). The model is evaluated against AC voltammetric data for ITIES formed between various aqueous electrolytes and an α,α,α-trifluorotoluene phase containing bis(triphenylphosphoranylidene)ammonium tetrakis(pentafluorophenyl)borate. The results support the presence of back-to-back diffuse layers within the electric double layer (EDL) and show that Helmholtz capacitance varies with interfacial charge density, which depends on the aqueous ions' ability to penetrate the mixed layer, their kosmotropic/chaotropic character, electrolyte concentrations in both phases, and the applied interfacial Galvani potential difference (Δ^w^_o_*ϕ*). These insights advance understanding of the EDL at polarisable liquid|liquid interfaces.

## Abstract 2

Extended Gouy–Chapman–Stern modelling reveals charge density dependent Helmholtz capacitance at polarisable liquid|liquid interfaces, governed by ion penetration into the mixed layer, electrolyte composition, and Galvani potential difference.

## Graphical abstract

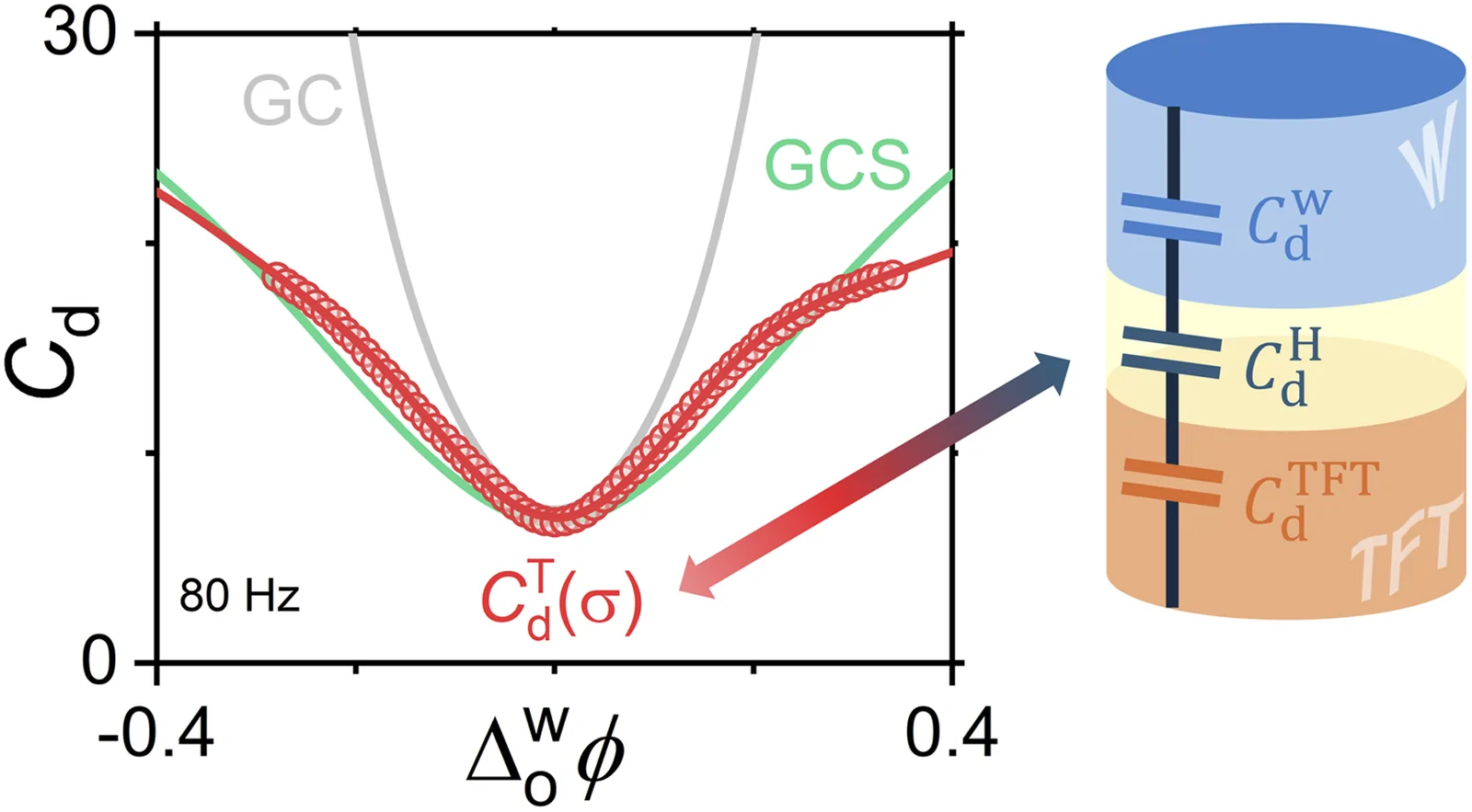

## Introduction

Electric potentials across liquid|liquid interfaces, as encountered in biological membranes and liquid-based batteries, play a central role in cellular metabolism, cell signalling, and energy storage.^1–4^ Consequently, understanding polarisable liquid|liquid interfaces is essential for elucidating the coupling between electrochemical reactions and electrochemical potential gradients, the kinetics of ion transfer processes across interfaces, and energy storage *via* interfacial capacitance.

Samec and co-workers developed the four-electrode potentiostatic configuration to study the Interface between Two Immiscible Electrolyte Solutions (ITIES).^5^ In this setup, two reference electrodes are employed to measure the interfacial potential difference across the liquid|liquid interface, while two auxiliary electrodes generate an electric field that changes the interfacial potential. This configuration provides improved control of the interfacial potential and enables more reliable and reproducible investigations of ion transfer processes.

At a metal|electrolyte interface, the interfacial potential difference can be modulated through electrode polarisation, which alters the Fermi level of the metal under potentiostatic control. In contrast, in the four-electrode ITIES configuration, the auxiliary electrodes generate an electric field that polarises both immiscible electrolyte phases, particularly the organic phase because of its higher electrical resistance. The difference in polarisation between the two phases establishes an interfacial potential difference that can drive ion transfer reactions or induce ion accumulation at the liquid|liquid interface.^6^

ITIES have been extensively studied over the past four decades^7–10^ using neutron^11,12^ and X-ray reflectivity,^13,14^ surface second harmonic generation,^15–18^ and Molecular Dynamics (MD) simulations.^19–21^ Structural information about the Electric Double Layer (EDL) at an ITIES can also be inferred from capacitance measurements at polarisable liquid|liquid interfaces.^22^ Experimental methods to measure capacitances include Electrochemical Impedance Spectroscopy (EIS),^22^ alternating current voltammetry (ACV),^23^ and electrocapillary curve analysis.^15,24,25^ Regardless of the method, interpreting differential capacitance *versus* interfacial Galvani potential difference (Δ^w^_o_*ϕ*) curves require a physicochemical model of the EDL and an equivalent circuit representation. Developing such models remains challenging due to the interplay of potential distributions, ion concentrations, solvent orientation, adsorption or electro-adsorption of neutral or charged species, and the complex dielectric properties of liquids at an ITIES.^23^ Consequently, the connection between experimental capacitance data and theoretical descriptions is still limited.

The classic Verwey–Niessen model describes the EDL at an ITIES as two back-to-back diffuse layers on either side of the liquid|liquid interface,^26^ with the potential distribution in the EDL defined by Gouy–Chapman theory.^27,28^ Adding the Stern modification leads to the Gouy–Chapman–Stern (GCS) model,^29^ which divides the EDL into three layers: two diffuse regions separated by an inner Helmholtz (or Stern/mixed) layer. In this modified Verwey–Niessen (MVN) model, the Helmholtz layer may be either ion-free and composed solely of solvent molecules,^30^ or a solvent layer partly penetrated by ions.^31^ Subsequent studies have examined how ion penetration into the inner layer, overlapping space-charge regions, interfacial ion distributions, ion association, dielectric properties of the aqueous and organic phases, and the applied Δ^w^_o_*ϕ*, influence capacitance.^32–48^ By contrast, Gschwend *et al.* proposed the discrete Helmholtz model, supported by experimental data at micro-liquid|liquid interfaces and MD simulations, which envisions correlated “face-to-face” ionic layers without diffuse regions.^15,19^ In a departure from the MVN structure, this model implies that the differential capacitance at an ITIES should be unaffected by the concentrations of aqueous and organic electrolytes.

Here, we apply GCS theory and follow the Grahame extension^49,50^ to include electrolytes with asymmetric charges and an inner Helmholtz layer, to model differential capacitance at the ITIES. A key innovation is an empirical equation that links the Helmholtz layer capacitance to the interfacial charge density. This dependence reflects (i) the dielectric saturation of solvent molecules under an intense local electric field that reduces the ability to screen surface charge, resulting in a reduction of the capacitance, (ii) the electrostatic repulsion of ions on the same side of the Helmholtz layer, and (iii) the solvent structure (particularly in the aqueous phase). Unlike traditional GCS theory, which assumes that the adsorption energy of ions is independent of the surface coverage,^51^ our model recognises that increasing electrostatic repulsion and finite ion size limit ion accumulation in the Helmholtz layer as Δ^w^_o_*ϕ* deviates from the Potential of Zero Charge (PZC). Stronger electric fields also reduce inner-layer permittivity,^52,53^ further lowering the Helmholtz capacitance as Δ^w^_o_*ϕ* is scanned away from the PZC.

Experimentally, we show that the range of potential where the behaviour of an ITIES is purely capacitive is narrow, making the width of the polarisable potential window (PPW) a critical parameter to optimise for accurate differential capacitance measurements. Using optimised PPWs, we measured differential capacitance profiles for aqueous electrolytes with symmetric (*z*_+_ = −*z*_−_) and asymmetric (*z*_+_ ≠ −*z*_−_) charges. These electrolytes vary in their tendency to penetrate and accumulate in the mixed layer, based on their relative solubilities in the organic phase and their ability to structure interfacial water (kosmotropic effects). For each ITIES system, electrolyte concentrations in both phases were systematically varied, and differential capacitance profiles were fit to our model. Analysis of fitting parameters, especially the dielectric constant of the organic diffuse layer, provides new insights into the EDL structure. Importantly, we deconvolute the Helmholtz contribution from the total differential capacitance, showing its dependence on the electrolyte's identity and concentration, as well as on the applied Δ^w^_o_*ϕ*. Finally, we demonstrate that when interfacial reactions occur upon polarisation of the ITIES, such as crystallisation, the model no longer accurately describes the measured differential capacitance behaviour.

## Results and discussion

### Mathematical model of the EDL at an ITIES

The capacitance of an ITIES as a function of the applied Δ^w^_o_*ϕ* is computed considering its equivalence to a circuit of three capacitors in series: two of which are related to the differential capacitance of the diffuse layers on either the aqueous side of the liquid|liquid interface (*C*^w^_d_), or its organic counterpart (*C*^o^_d_), and the third is related to the Helmholtz layer differential capacitance (*C*^H^_d_). The total (or composite) interfacial differential capacitance (*C*^T^_d_) per unit area is given by:1
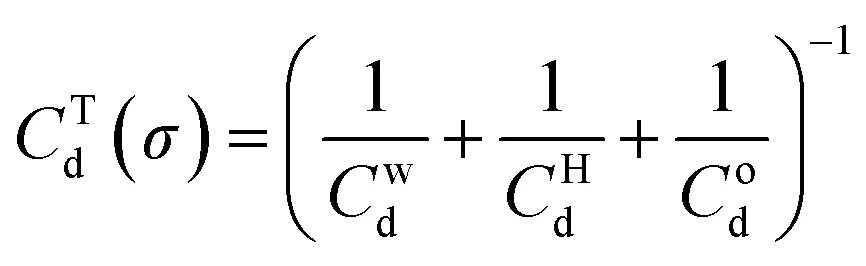
and Δ^w^_o_*ϕ*^T^ is defined as:

2 Δ^w^_o_*ϕ*^T^(*σ*) = Δ*ϕ*^w^(*σ*) + Δ*ϕ*^H^(*σ*) + Δ*ϕ*^o^(*σ*) = (Δ*ϕ*^b,w^ − Δ*ϕ*^b,o^)

where Δ^w^_o_*ϕ* and Δ*ϕ*^o^ are the potential drops across the diffuse layers on the aqueous and organic sides of the EDL, respectively, and Δ*ϕ*^H^ is the potential drop across the Helmholtz layer. In turn Δ*ϕ*^w^ = (*ϕ*^b,w^ − *ϕ*^H,w^), Δ*ϕ*^o^ = (*ϕ*^H,o^ − *ϕ*^b,o^), and Δ*ϕ*^H^ = (*ϕ*^H,w^ − *ϕ*^H,o^), where *ϕ*^H,w^ and *ϕ*^H,o^ are the coordinates of the aqueous and organic Outer Helmholtz Planes (OHPs), respectively, see Fig. 1. In this circuit, all capacitors are connected in series. Therefore, each capacitor can be associated with the equivalent electrical charge accumulated at the interface, and this charge value, divided by the interfacial area, corresponds to the interfacial charge density, *σ*. That is, upon polarisation by an applied Δ^w^_o_*ϕ*, the liquid|liquid interface will be capable of holding a charge density *σ*. As illustrated in Fig. 1, when the interface is positively polarised, cations will accumulate on the aqueous side (+*σ*), and anions on the organic side of the interface (−*σ*). Conversely, when the interface is negatively polarised, the opposite effect will occur.

**Fig. 1:**
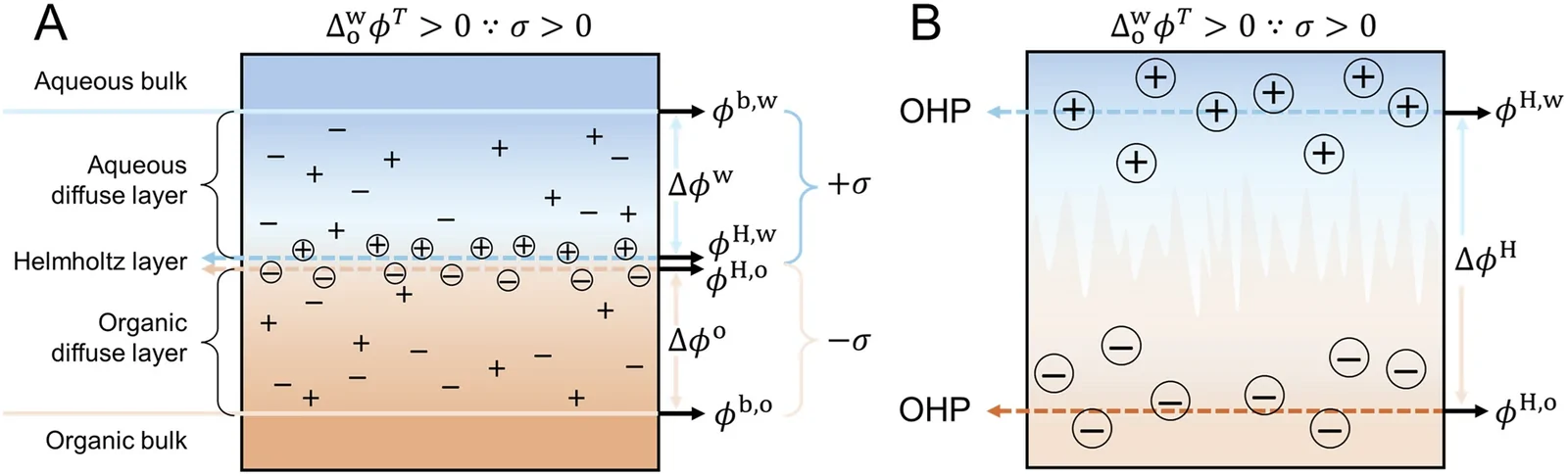
Schematic representation of the structure of the electric double layer (EDL) of a polarisable interface between two immiscible electrolyte solutions (ITIES). The EDL consists of three layers: (A) two back-to-back diffuse layers on either side of the liquid|liquid interface and an inner Helmholtz layer. A zoom-in of the Helmholtz layer in (B) shows the Outer Helmholtz Planes (OHPs) facing each diffuse layer. In the schemes in (A) and (B), the accumulation of charge at the liquid|liquid interface is depicted for a positive applied interfacial Galvani potential difference (Δ^w^_o_*ϕ* > 0 V). This EDL structure is used to formulate the mathematical model required to simulate the differential capacitance as a function of the applied Δ^w^_o_*ϕ* at an ITIES. The potential differences, Δ*ϕ*^w^ = (*ϕ*^b,w^ − *ϕ*^H,w^), Δ*ϕ*^o^ = (*ϕ*^H,o^ − *ϕ*^b,o^), and Δ*ϕ*^H^ = (*ϕ*^H,w^ − *ϕ*^H,o^), and their coordinates are shown in each panel.

If the stored interfacial charge density is known, the potential drop at each capacitor can be computed. Hence, the simulation starts with a unidimensional vector of stored electric charge per unit area. The list used has 100 points distributed in a parabolic fashion between ∼±8 × 10^−2^ C m^−2^. The vertex of this parabola is set at zero; consequently, Δ^w^_o_*ϕ*^T^(0) is the PZC in the (Δ^w^_o_*ϕ* − Δ^w^_o_*ϕ*_PZC_) scale. The range of charge densities employed is in line with the values typically observed from differential capacitance profiles measured by ACV, and other surface charge density reports,^15^ at the ITIES. It is worth noting that, even in the absence of specific adsorption, Δ^w^_o_*ϕ*_PZC_ is rarely at exactly 0 V on the Galvani scale. Instead, from a practical viewpoint, it is equivalent to the applied Δ^w^_o_*ϕ* either at the maximum of an electrocapillary curve measurable by the pendant drop imaging method^54^ or, as is the case in this article, at the minimum of a differential capacitance curve.^55^ Knowing the stored charge, the potential drop at each of the back-to-back diffuse layers is calculated by solving their respective Poisson–Boltzmann equation (see Section S1.1), for example, for the organic diffuse layer:3
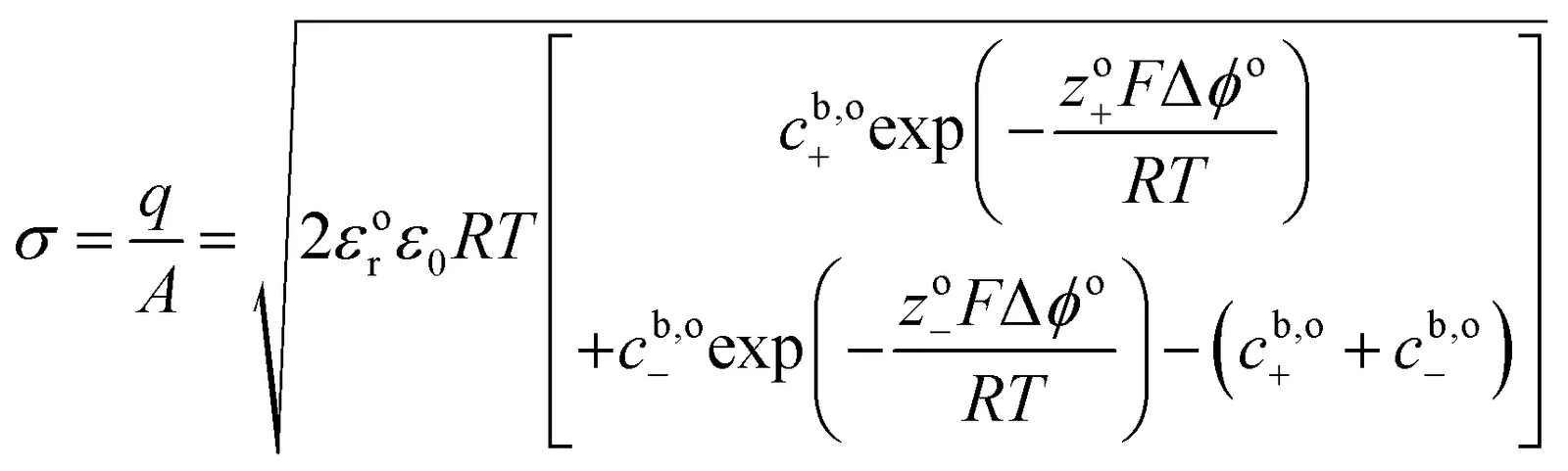
where Δ*ϕ*(*σ*) is the potential drop function of the respective diffuse layer, *c*^b^_+_ (*c*^b^_−_) and *z*_+_ (*z*_−_) are the bulk concentrations and charge values, respectively, of the cations (anions) in each phase, *ε*_r_ is the relative permittivity of the solvent where the respective diffuse layer is located, and the other terms have their usual meaning. Note that the relation between Δ*ϕ*(*σ*) and *σ* can be simplified and solved analytically for symmetrically charged aqueous or organic electrolytes (*z*_+_ = −*z*_−_ and *c*^b^_+_ = *c*^b^_−_, see Section S1.2). Nonetheless, when studying asymmetric electrolytes in either phase, a numerical method was implemented to find the potential drop. In this instance, Δ*ϕ*(*σ*) was calculated using an optimisation program. In our case, we employed Solver, the add-in tool available in Excel or OpenOffice. Subsequently, the function of the differential capacitance of each diffuse layer per unit area (*C*_d_(Δ*ϕ*,*σ*)) is obtained by differentiation, as shown in the case of the diffuse organic layer in (4):4
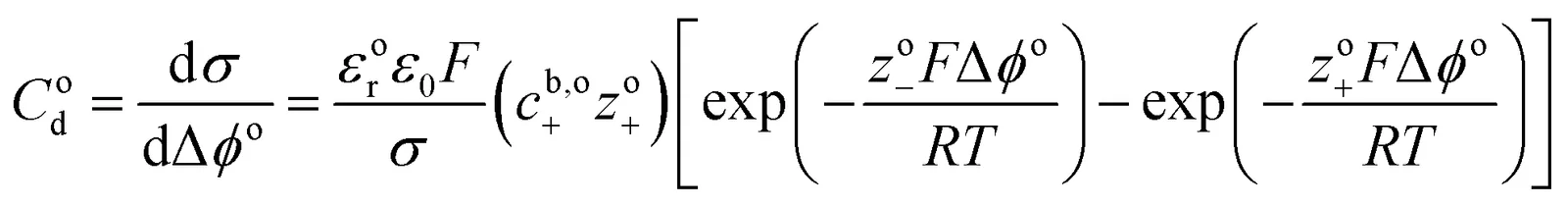


For the simulation of *C*^H^_d_, the following three approaches were analysed, where the Helmholtz layer capacitance: (i) does not exist or is much higher than the diffuse layer capacitances, (ii) is a constant, according to the GCS model, and (iii) decreases at potentials far away from the PZC and is thus dependent on the applied Δ^w^_o_*ϕ*. That is, the Helmholtz layer is defined as the region within the mixed layer at the liquid|liquid interface, where the electrolyte does not follow the ion distribution predicted by the Poisson–Boltzmann equations, but rather exhibits the semi-empirical behaviour described by eqn (5):

5 *C*^H^_d_(*σ*) = *α* exp(−*β*|*σ*|) + *γ*

This equation was chosen to simulate *C*^H^_d_ based on the following observations. First, the influence of *C*^H^_d_ must be negligible around the PZC. This implies that *C*^H^_d_ has to be significantly higher than *C*^o^_d_ at potentials close to the PZC. For this reason, we assumed an exponential decrease of *C*^H^_d_ as a function of the absolute value of the interfacial charge density. At potentials far from the PZC, *C*^o^_d_ becomes very large, and therefore, the total differential capacitance is predominantly determined by *C*^H^_d_. Second, we postulated that *C*^H^_d_ should decrease as Δ^w^_o_*ϕ* deviates further from the PZC. This behaviour is attributed to the electrostatic repulsion between the accumulated anions on both sides of the interfacial Helmholtz capacitor, as well as to the finite size of ions, which cannot be treated as point charges, and to the solvent polarisation. Such behaviour resembles adsorption processes in which the adsorption energy changes with surface coverage due to repulsive interactions, as described by the Frumkin isotherm. In summary, eqn (3) and (4) determine the capacitance behaviour near the PZC, whereas eqn (5) becomes relevant at potentials far from the PZC. Finally, the parameters *α*, *β* and *γ* are empirical constants (see Section S1.3).

The Helmholtz capacitance is generally assumed to be constant at the metal electrode|electrolyte interface in simple classical models. However, it often exhibits a distinct peak-shaped profile or asymmetry around the PZC. At the PZC, water molecules at the electrode surface are randomly oriented. As the electrode potential moves away from the PZC, the increasingly intense local electric field induces dielectric saturation of solvent molecules in the Helmholtz layer, leading to their full polarisation. Consequently, their ability to screen surface charge decreases, resulting in a reduction of the capacitance.^56–59^Eqn (5) was introduced to reproduce the behaviour described above, typically observed at metal|electrolyte interfaces, but in the case of a liquid|liquid interface.

The best fit was found by minimising the Root Mean Squared Error (RMSE) using Solver. Values of fundamental constants and fixed parameters used to run the simulation are listed in Table S1 (Section S1.4). The fittings were carried out on the (Δ^w^_o_*ϕ* − Δ^w^_o_*ϕ*_PZC_) scale, *i.e.*, Δ^w^_o_*ϕ*_PZC_ was determined for each ITIES configuration studied (Table S2) and subsequently set to 0 V (Section S1.5). As discussed in more detail in the Materials and methods (Section S2), to obtain high-quality, reproducible experimental data, cyclic voltammograms and differential capacitance curves for a “reference biphasic system” were obtained to diagnose if contaminants were present (Fig. S1), and signals described in Table S3 are typical of a contaminated biphasic system (Section S2.5).

### Optimising the polarisable potential window for recording differential capacitance profiles at the ITIES

Experimentally, the capacitive component of an electrochemical system can be calculated from impedance measurements. At a polarisable liquid|liquid interface, the impedance spectrum (from EIS) can be interpreted through equivalent circuit models, enabling the capacitance to be rationalised. In ACV, the frequency must be chosen such that the mathematical relationship between capacitance and impedance is defined. The ideal capacitor approximation *C*_d_ = (−*Z*_j_*f*2π)^−1^, is commonly used to calculate differential capacitance profiles from ACV, where *Z*_j_ is the imaginary component of the impedance and *f* is the frequency (in Hz). This expression is also used to estimate the reactance of the capacitor^60^ or calculate complex capacitance for transforming impedance spectra into capacitance spectra.^61,62^ However, applying this transformation in ACV does not guarantee that the resulting signal reflects the true interfacial capacitance, free from the influence of ion transfer reactions, which is herein referred to as the faradaic ion transfer contribution. In practice, capacitance-like profiles derived from ACV can still be useful, for example, in quantifying tetraalkylammonium ions at the ITIES, even though ion transfer generates faradaic currents.^63^ Thus, to understand and model the intrinsic differential capacitance, the design of the ACV method must be guided by insights from CV and EIS.

A key consideration is the PPW. If the applied Δ^w^_o_*ϕ* extends too positive or negative, ion transfer occurs across the interface, compromising capacitive behaviour. A schematic representation of the general configuration of the various four-electrode electrochemical cells studied herein using a polarisable aqueous|TFT interface is provided in Fig. 2A. The electrochemical measurements presented in Fig. 2B–E are for an ITIES prepared with calcium chloride (CaCl_2_) in the aqueous phase and bis(triphenylphosphoranylidene)ammonium tetrakis(pentafluorophenyl)borate (BATB) electrolyte in an α,α,α-trifluorotoluene (TFT) organic phase. Cyclic voltammograms were recorded with the PPW delimited during the CV cycle by current cutoffs of either ±1 or ± 2 µA cm^−2^, respectively (Fig. 2B). The PPWs determined by CV at each current cutoff were then used to obtain differential capacitance profiles by ACV measurements (Fig. 2C). The narrowness of the potential range of the purely capacitive region at the ITIES is evident: even at a cutoff of ±2 µA cm^−2^, deviations appear as plateaus in the differential capacitance profile (150–200 mV wide), followed by a significant divergence of current due to faradaic ion transfer (Ca^2+^ at the positive edge of the PPW, Cl^−^ at the negative edge).

**Fig. 2:**
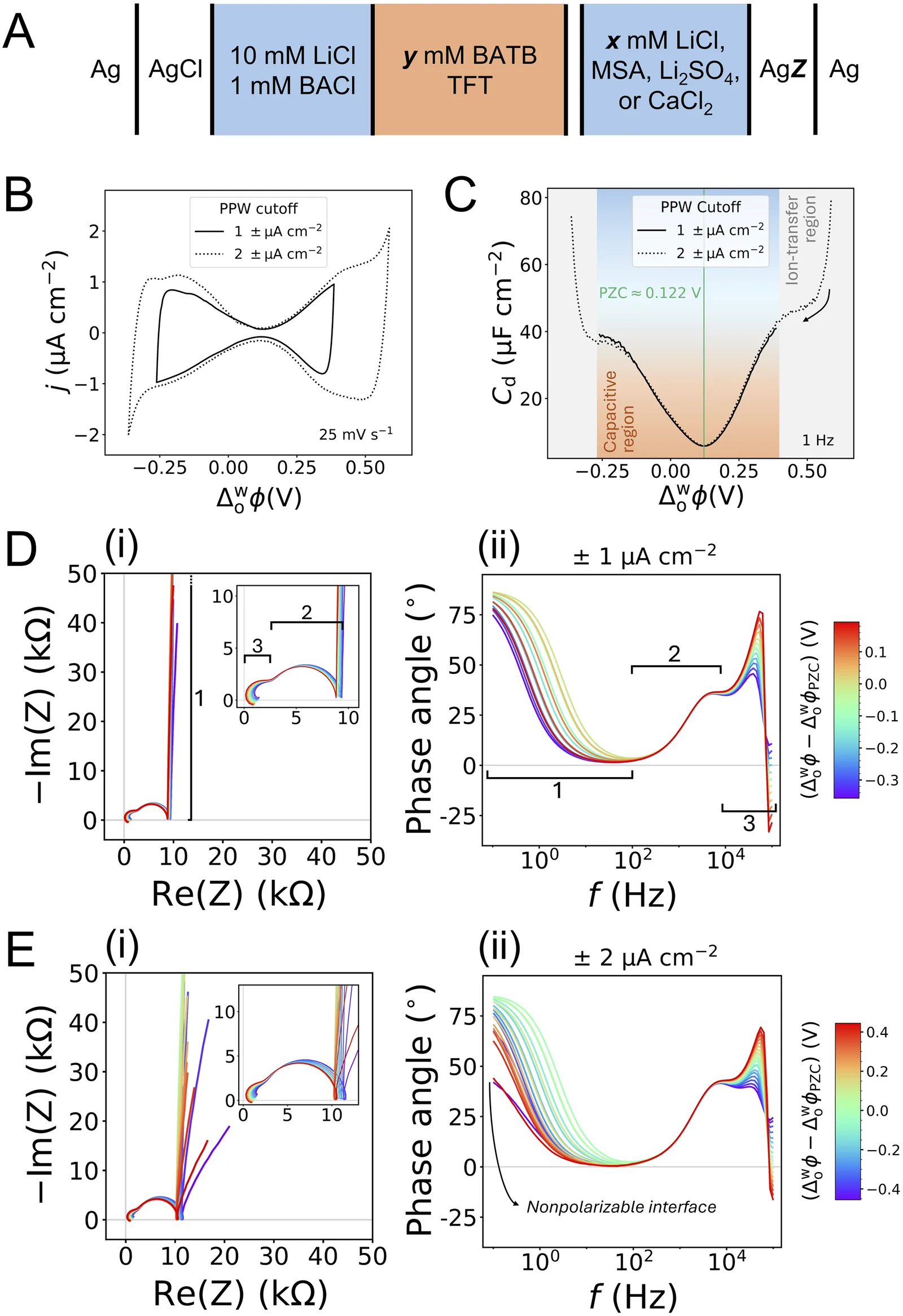
Effect of the width (or potential range) of the polarisable potential window (PPW) on the AC electrochemical performance of a polarisable liquid|liquid interface. (A) Schematic representation of the general configuration of the various four-electrode electrochemical cells studied using a polarisable aqueous|α,α,α-trifluorotoluene (TFT) interface in this article. The different concentrations of the electrolyte in each phase are represented by *x* and *y*, and *Z* is the anion of the aqueous electrolyte investigated. The data shown in (B)–(E) were obtained using the cell configuration described in (A) with 1.1 mM CaCl_2_ aqueous electrolyte and 1.0 mM BATB organic electrolyte (*i.e.*, *x* = 1.1, *y* = 1.0, and *Z* = Cl). (B) Cyclic voltammograms with the width of the PPW delimited by a current cutoff of either ±1 or ±2 µA cm^−2^. The scan rate used was 25 mV s^−1^. (C) Comparison of differential capacitance profiles obtained from AC voltammetry (ACV) measurements at a frequency of 1 Hz and 10 mV of amplitude. The scan direction was from the positive to the negative ends of the PPW, as indicated by the arrow. (D and E) Nyquist and phase angle Bode plots prepared from potentiodynamic electrochemical impedance spectroscopy (PDEIS) measurements. PDEIS spectra were measured across a frequency range of 0.1 Hz to 0.1 MHz, with an AC amplitude of 10 mV, every 0.05 V across the width of each PPW investigated. The ACV and PDEIS measurements were carried out using the PPWs determined by CV in (B) at current cutoffs of either ±1 or ±2 µA cm^−2^ as indicated. The spectra in the Nyquist and phase angle Bode plots are on the (Δ^w^_o_*ϕ* − Δ^w^_o_*ϕ*_PZC_) scale, described in the Materials and methods (Section S2.3), as per the colored sidebars.

Potentiodynamic electrochemical impedance spectroscopy (PDEIS) measurements further demonstrate this narrow capacitive region at an ITIES. Nyquist and phase angle Bode plots show that widening the PPW, by increasing the current cutoffs from ± 1 to ± 2 µA cm^−2^ (Fig. 2D–E), causes the interface to deviate from ideal resistor-capacitor (RC) behaviour at low frequencies (0.1–100 Hz), as mass transport contributions emerge. This is seen as a tilt in Nyquist plots (compare Fig. 2D(i) and E(i)) and decreased phase angles at low frequency in Bode plots (Fig. 2D(ii) and E(ii)) for applied Δ^w^_o_*ϕ* at the edges of the PPW. Therefore, to model differential capacitance profiles using thermodynamics, capacitance must be measured within a PPW where the ITIES remains fully polarisable.

Unlike PDEIS, ACV probes a single frequency, typically between 0.1–100 Hz at polarisable liquid|liquid interfaces^23,64^ (region 1 in Fig. 2D(i) and (ii)). Frequency selection depends on both the equivalent circuit and capacitance dispersion caused by dielectric relaxation,^23,62^ see Sections S3.1 and S3.2. The standard assumption is that the EDL behaves as an ideal capacitor within an RC series circuit, giving frequency-independent capacitance. In practice, some ITIES approximate this behaviour below 100 Hz. However, this mathematical transformation can yield translated capacitance profiles because the calculated magnitude varies with frequency. As we reported previously,^23,63^ when reversible ion transfer occurs within the PPW, higher frequencies (≥80 Hz) suppress the faradaic impedance, maximising the capacitive response. Lower frequencies (<10 Hz) enhance ion transfer features, improving peak resolution. By selecting appropriate frequencies (≥80 Hz), capacitance values that are more representative of the intrinsic interface can be obtained, even at the edges of the PPW.

At higher frequencies (>100 Hz), additional features, such as semicircles or loops, appear in the Nyquist plot.^22,65,66^ In the CaCl_2_|TFT(BATB) cell (Fig. 2D and E), two coupled charge-transfer-like events are observed: one stable semicircle (10^2^–10^4^ Hz; region 2) and another potential-dependent feature (10^4^–10^5^ Hz; region 3), suggesting that the resistance to charge transfer of this process is proportionally dependent on the potential. The potential-dependent feature is coupled to an inductor, as indicated by imaginary impedance values greater than zero and corresponding negative phase angles at frequencies greater than 8 × 10^4^ Hz. Such high-frequency behaviour has been attributed by Samec *et al.* to parasitic coupling between electrodes and potentiostat inputs.^22,65,66^ Although this observation is outside the scope of this paper, we demonstrate that electrolyte composition affects these features (Section S3.3) and provide evidence of parasitic coupling between electrodes and potentiostat inputs (Section S3.4). This implies that both instrumental artefacts and genuine interfacial processes contribute to the observed semicircles and loops.

### Influence of the nature and concentration of the electrolytes on differential capacitance profiles at the ITIES

For each four-electrode electrochemical cell studied (using LiCl, Li_2_SO_4_, or methanesulfonic acid (MSA) as the aqueous electrolyte), ACV measurements were carried out within the PPWs determined from CV at current cutoffs of ±1 µA cm^−2^ (Section S4). Increasing either the aqueous or the organic electrolyte concentration consistently enhanced the differential capacitance across the entire profile (Fig. 3), providing clear evidence of the presence of diffuse layers within the EDL structure at polarisable aqueous|TFT interfaces. The differential capacitance was more sensitive to changes in BATB concentration in the organic TFT phase than to changes in aqueous electrolyte concentration. Specifically, a two-order-of-magnitude increase in aqueous electrolyte concentration produced changes comparable to those caused by only a few millimolar increases in BATB concentration (Fig. 3). This disparity arises because, under an applied Δ^w^_o_*ϕ*, the ohmic drop is much larger in the organic phase than in the aqueous phase, because *ε*^w^_r_ ≫ *ε*^o^_r_.^23^ As a result, the organic phase experiences a stronger electric field, increasing its polarisability and making the capacitance curves more sensitive to the concentration of the organic electrolyte.

**Fig. 3:**
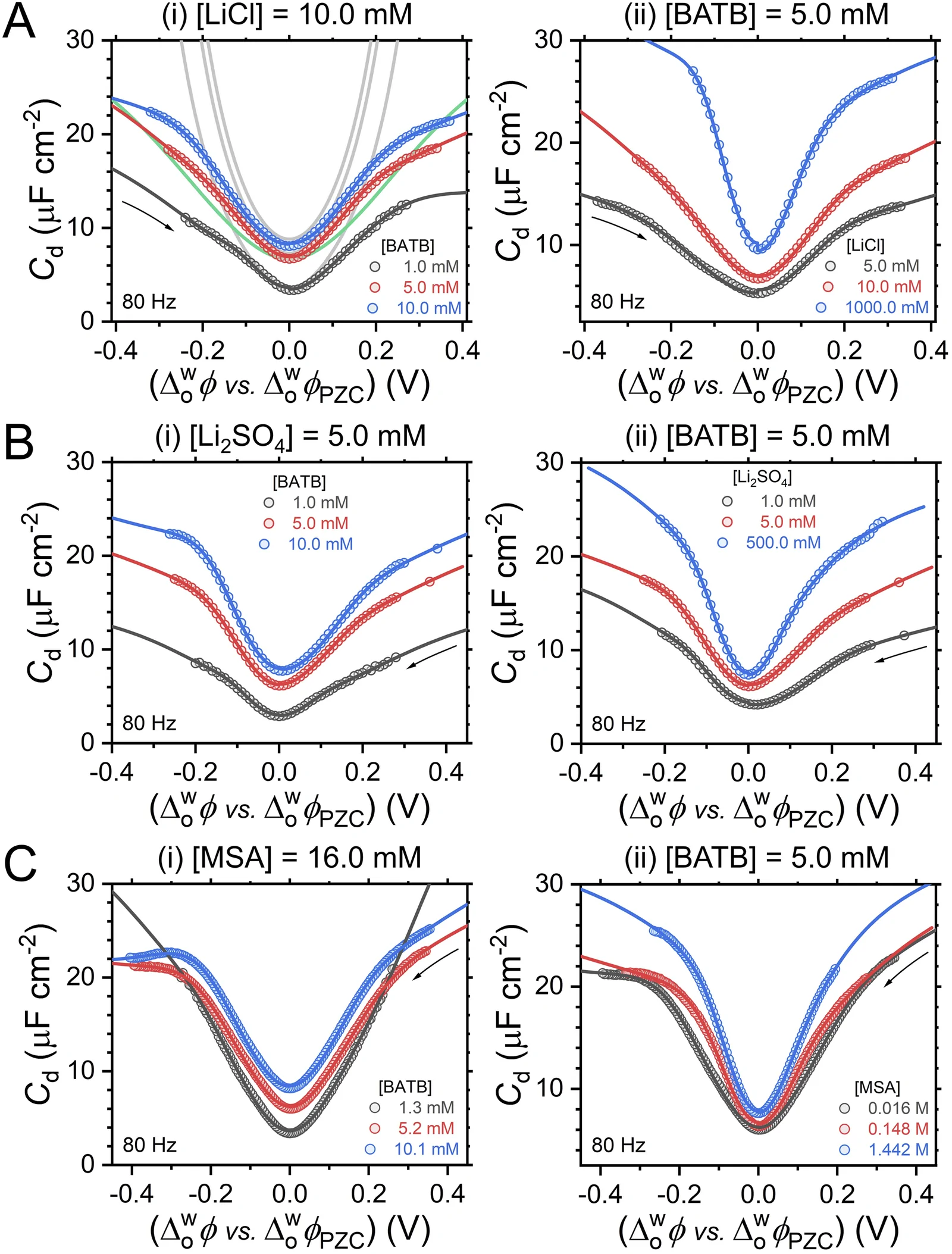
Influence of the nature and concentration of the electrolytes on differential capacitance profiles at the ITIES. Comparison of differential capacitance profiles obtained from ACV for ITIES prepared with aqueous electrolyte solutions of either (A) LiCl, (B) Li_2_SO_4_, or (C) MSA. The organic electrolyte was BATB in all cases. The concentrations of the various aqueous electrolytes and BATB organic electrolyte are indicated in each panel. The grey lines in (A) show the differential capacitances calculated with Gouy–Chapman theory assuming a model composed of two back-to-back diffuse layers. The green line in (A) shows the capacitances with Gouy–Chapman–Stern (GCS) theory, with a constant value for the Helmholtz (or mixed) layer capacitance (*C*^H^_d_) of 40 µF cm^−2^. The solid lines in all panels show the capacitances calculated with GCS theory, using three capacitors in series, and assuming a model composed of two back-to-back diffuse layers and a *C*^H^_d_ that changes with the interfacial charge density (*σ*). ACV measurements for each ITIES were carried out using the four-electrode electrochemical cell configuration described in Fig. 2A at a frequency of 80 Hz and an amplitude of 10 mV. The arrow indicates the scan direction in each case. The measurements were carried out using the PPWs determined by CV in Section S4 with current cutoffs of maximum ±1 µA cm^−2^. The fittings were carried out on the (Δ^w^_o_*ϕ* − Δ^w^_o_*ϕ*_PZC_) scale as described in the Materials and methods.

Application of the Gouy–Chapman theory, which treats the ITIES as two back-to-back diffuse layers, allows prediction of differential capacitances near the PZC (solid grey lines in Fig. 3A(i) for a cell with 10.0 mM LiCl aqueous and *x* mM BATB organic electrolyte). This indicates that the Helmholtz layer is negligible at such potentials, or alternatively, that *C*^H^_d_ is much larger than *C*^w^_d_ and *C*^o^_d_. However, Gouy–Chapman theory predicts perfectly parabolic-shaped differential capacitance curves, *i.e.*, symmetric around the PZC and tending to infinity at the edges, which differs from experimental observations. Experimentally, differential capacitance curves are sometimes symmetric but never tend to infinity within the PPW. Moreover, asymmetry becomes increasingly pronounced at higher aqueous electrolyte concentrations (Fig. 3A(ii)–C(ii)), especially when lithium salts are present. Thus, Gouy–Chapman theory alone cannot adequately describe the experimental differential capacitance behaviour at the ITIES.

Following Stern's proposal for solid electrode|electrolyte interfaces,^29^ better agreement is obtained between experimental and calculated data when *C*^H^_d_ is introduced and set to a constant value (GCS theory) as a function of the applied Δ^w^_o_*ϕ*. For instance, the solid green line in Fig. 3A(i) shows the GCS calculation with *C*^H^_d_ = 40 µF cm^−2^. In this framework, lowering *C*^H^_d_ flattens the capacitance profile, while increasing it causes convergence toward the Gouy–Chapman prediction (solid grey lines), see Section S5.1 and Fig. S6. Although Stern's modification improves the fit, the GCS model still cannot reproduce key experimental features such as the plateau regions at the flanks of the PPW.

To address these shortcomings, we propose a modified GCS model in which *C*^H^_d_ is treated as a function of the interfacial charge density, as expressed in eqn (5). This approach significantly improves the correlation between experimental and theoretical differential capacitance profiles (solid lines in each panel in Fig. 3). Details on parameter uniqueness and the statistical analysis of the fits for each ITIES are presented in Sections S5 and S6, respectively.

### Deconvolution of the Helmholtz layer differential capacitance from the total differential capacitance at an ITIES

Additional insights into the electrolyte behaviour are revealed through the Helmholtz layer differential capacitance curves (Fig. 4). These semiempirical profiles display a peak-shaped dependence on the applied Δ^w^_o_*ϕ* (d*C*^H^_d_(*σ*)/d*σ* < 0). The peak-shape arises from steric effects combined with enhanced electrostatic repulsion of ions in the inner layer, and a decrease in its relative permittivity as Δ^w^_o_*ϕ* is scanned away from the PZC,^67^ as parameterised in eqn (5). To our knowledge, the only previous deconvolution of the Helmholtz layer differential capacitance (*C*^H^_d_) from the total differential capacitance (*C*^T^_d_) at an ITIES was carried out by Samec *et al.* from electrocapillary curve data.^25^ Fully in line with our observations in Fig. 4, Samec *et al.* reported that *C*^H^_d_ has a maximum at zero surface charge (*i.e.*, at the PZC) for a similar interface, LiCl in water and tetrabutylammonium tetraphenylborate in nitrobenzene, and attributed this to the change in the surface dipole orientation with the electric field.^25^ Similar peak features have been observed previously at single-crystal electrode|electrolyte interfaces,^59,68,69^ particularly at electrolyte concentrations ≥5 mM, and attributed to water-induced polarisability in the Helmholtz layer.

**Fig. 4:**
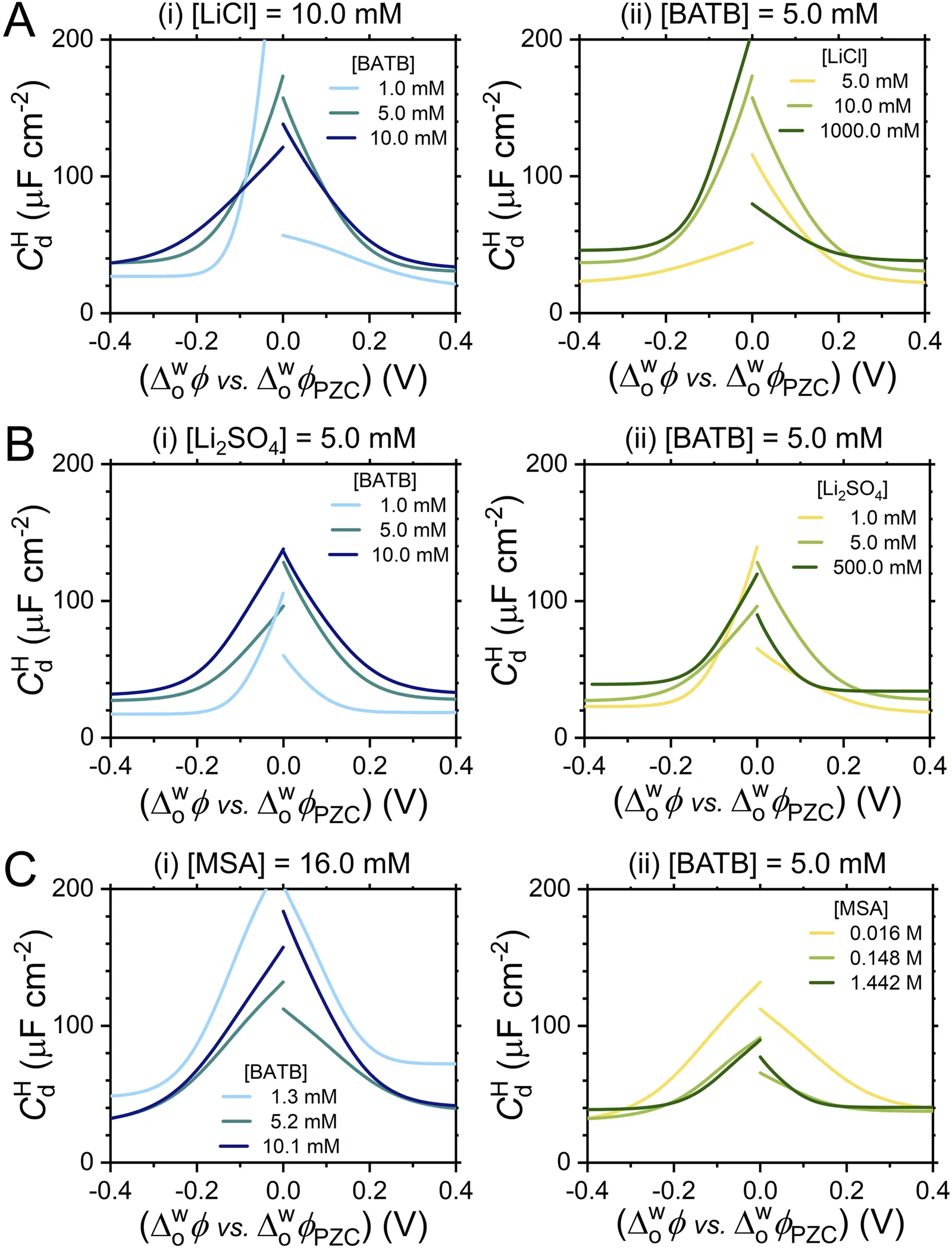
Deconvolution of the Helmholtz layer differential capacitance from the total differential capacitance at an ITIES. Comparison of Helmholtz layer differential capacitance (*C*^H^_d_) curves simulated using eqn (5) for the data shown in Fig. 3 for ITIES prepared with aqueous electrolyte solutions of either (A) LiCl, (B) Li_2_SO_4_, or (C) MSA. The organic electrolyte was BATB in all cases. The concentrations of the various aqueous electrolytes and BATB organic electrolyte are indicated in each panel. The *C*^H^_d_ curves are plotted on the (Δ^w^_o_*ϕ* − Δ^w^_o_*ϕ*_PZC_) scale as described in the Materials and methods.

The capacitance contributions from the Helmholtz and diffuse layers can exceed the total equivalent capacitance of the interface by an order of magnitude at their maxima (Fig. 4). According to the model, such increase is associated with the potential decay length within each layer, rendering them more compact. For the diffuse layers, this effect is reflected in the Debye length, which shortens with higher bulk electrolyte concentrations or elevated interfacial ion concentrations near the PPW edges. In contrast, the Helmholtz layer thickness, already on the order of atomic dimensions (<10 Å), shrinks further near the PZC, becoming practically negligible.^48^ Therefore, the capacitance of each interface layer can suddenly increase, since it is normalised to the charge space of the component it represents. Identical magnitudes of the inner-layer differential capacitance have been deconvoluted across different metal|electrolyte interfaces.^70–72^

The proposed physical model does not allow the accurate determination of *C*^H^_d_ at potentials close to the PZC. Therefore, only data obtained at potentials more than 100 mV away from the PZC should be considered meaningful. Nevertheless, the *C*^H^_d_ values obtained at potentials less than ±100 mV from the PZC are remarkably close to those reported by Le *et al.* for the *C*^H^_d_ of the Pt(111)|water interface (see Fig. 5of ref. 59).

**Fig. 5:**
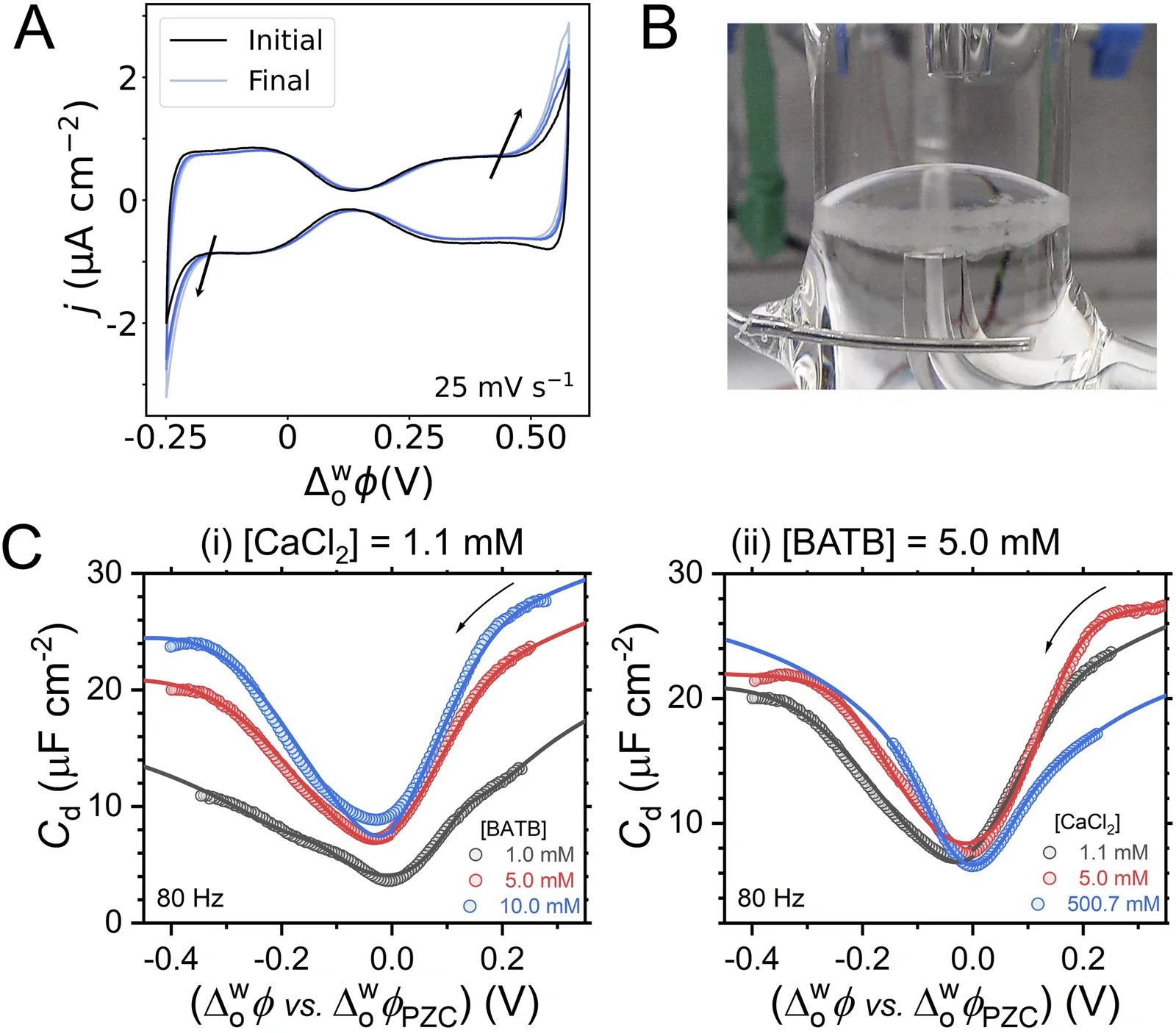
Attempts to model differential capacitance profiles for ITIES prepared with electrolytes that undergo interfacial chemical reactions upon polarisation of the liquid|liquid interface. (A) Evolution of the CV signals after applying each AC method to the cell with 0.5 M CaCl_2_ aqueous electrolyte and 5.0 mM BATB organic electrolyte, setting an initial current cutoff of ± 2 µA cm^−2^. (B) Photograph of the formation of CaCl_2_ crystals at the three-phase contact point between the aqueous|TFT interface and the glass wall of the four-electrode electrochemical cell described in (A) after CV and ACV cycling. (C) Differential capacitance profiles obtained from ACV for ITIES prepared with CaCl_2_ aqueous electrolyte and BATB organic electrolyte of various concentrations, as indicated in each panel. The solid lines show the capacitances calculated with GCS theory, using three capacitors in series, and assuming a model composed of two back-to-back diffuse layers and a *C*^H^_d_ that changes with the interfacial charge density (*σ*). ACV measurements were carried out using the four-electrode electrochemical cell configuration described in Fig. 2A, and the PPW was determined by CV setting a current cutoff of ± 1 µA cm^−2^. The arrow indicates the scan direction in each AC voltammogram.

The magnitude and potential dependence of *C*^H^_d_ varies with electrolyte identity and concentration. In early capacitance reports, this dependence was attributed to specific adsorption of ions.^72,73^ However, for the polarisable liquid|liquid interfaces modelled herein, this is not the most accurate causality. During polarisation, the ITIES is not at thermodynamic equilibrium; some portion of ions transfer across the liquid|liquid interface at all Δ^w^_o_*ϕ* applied.^23^ For this reason, concentration dependence is plausible in the Helmholtz layer, thereby modulating interfacial dielectric properties.

For LiCl and Li_2_SO_4_ aqueous electrolytes, *C*^H^_d_ tends to increase with electrolyte concentration when the potential is scanned far from the PZC (Fig. 4A and B). Hence, the magnitude of *C*^H^_d_ at the plateaus is mainly influenced by the population of interfacial charge carriers. For example, at positive potentials *vs.* the PZC (Δ^w^_o_*ϕ* > 0.2 V *vs.* Δ^w^_o_*ϕ*_PZC_), where aqueous cations dominate the EDL, the trend and magnitude of *C*^H^_d_ is similar for both electrolytes (Fig. 4A(ii) and B(ii)), as a result of the equivalent change in Li^+^ concentration for both systems. In general, the extent of *C*^H^_d_ for LiCl is bigger than for Li_2_SO_4_. This increase is associated with enhanced mutual solubility of the aqueous and organic phases (discussed in more detail *vide infra*),^74^ which raises the polarisability of the mixed region and thus increases capacitance. However, at negative potentials, where anions dominate, if the concentration of anions is equivalent, *C*^H^_d_ is larger for SO_4_^2−^ than for Cl^−^ (see profiles for 5.0 mM in Fig. 4A(ii) and B(ii)). This is likely due to Cl^−^ crossing the interface more easily, while SO_4_^2−^ tends to accumulate in the aqueous Helmholtz plane, shrinking the inner-layer charge space.

The MSA system behaves differently. Here, *C*^H^_d_ shows no consistent dependence on the organic electrolyte concentration around the PZC (Fig. 4C(i)). Instead, the peak broadens, and the drop in capacitance with potential becomes more pronounced as the concentration decreases. Furthermore, increasing the electrolyte concentration reduces *C*^H^_d_ (Fig. 4C(i) and (ii)), with the profiles converging above approximately 100 mM and 5 mM for the aqueous and organic electrolytes, respectively. This effect is consistent with the combination of the kosmotropic nature of methanesulfonate anions (MS^−^) and the finite capacity of the liquid|liquid interface to accommodate them. MSA enhances water structuring and hydrogen bonding interactions at higher concentrations,^75,76^ limiting the polarisability of molecules at the interface and thus diminishing *C*^H^_d_. In addition, the hydration free energy of MS^−^ is smaller than that of SO_4_^2−^,^77,78^ favouring partial dehydration and enrichment within the inner layer. Consequently, the concentration of MS^−^ in the Helmholtz layer increases only up to a saturation limit imposed by the finite capacity of the interfacial region, with additional anions accumulating in the diffuse layer. By contrast, SO_4_^2−^ remains predominantly hydrated and exhibits a weaker tendency to partition into the inner layer. Consequently, increasing the bulk concentration allows additional SO_4_^2−^ anions to be accommodated at the Helmholtz plane, increasing *C*^H^_d_.

Our earlier simulations of ion distributions at aqueous|TFT interfaces,^79,80^ based on a modified Poisson–Boltzmann framework with Bikerman's volume-exclusion correction,^81^ provide further context. These calculations predict sharp increases in interfacial ion concentrations as Δ^w^_o_*ϕ* is scanned ±0.2 V from the PZC. For instance, at +0.4 V *vs.* Δ^w^_o_*ϕ*_PZC_, the interfacial H^+^ concentration reaches ∼1 M, far above the 5 mM bulk H_2_SO_4_ concentration.^79^ Under such conditions, GCS theory collapses to a “discrete Helmholtz model” because the diffuse-layer contributions (*C*^w^_d_ and *C*^o^_d_) become negligible (in other words, the reciprocals of the capacitance of the diffuse layers in eqn (1) become close to zero). This parallels observations at solid electrode|electrolyte interfaces, where diffuse-layer effects vanish at electrolyte concentrations ≥1 M.^57^ In the experimental differential capacitance profiles (Fig. 3), this discrete Helmholtz behaviour manifests as plateaus at the PPW edges, spanning ∼150–200 mV just before the faradaic ion transfer regions (Fig. 2C). Within these plateaus, *C*^H^_d_ dominates the total capacitance. For LiCl and Li_2_SO_4_ systems (Fig. 4A and B), plateaus are most pronounced under conditions of low BATB concentration and high aqueous electrolyte concentration, where *C*^H^_d_ becomes independent of the applied Δ^w^_o_*ϕ*, consistent with discrete Helmholtz behaviour.

Analysis of the deconvoluted diffuse-layer differential capacitances (*C*^w^_d_ and *C*^o^_d_) on the (Δ^w^_o_*ϕ* − Δ^w^_o_*ϕ*_PZC_) scale (see Section S7) shows that at very high aqueous electrolyte concentrations, *C*^w^_d_ becomes nearly constant and much larger than both *C*^o^_d_ and *C*^H^_d_. In this regime, the differential capacitance of the interface is mainly influenced by *C*^H^_d_ at the PPW edges (as described previously) and by *C*^o^_d_ near the centre.

### Dielectric properties at the aqueous|TFT interface

In Gouy–Chapman theory, bulk permittivity values are used to calculate the electrochemical properties of the aqueous and organic diffuse layers at the liquid|liquid interface. In practice, however, the permittivity of the organic diffuse layer must differ from that of the bulk organic phase. Consistent with this expectation, higher relative permittivity values for TFT (*ε*_TFT_) in the organic diffuse layer than those reported for pure TFT were required to achieve a satisfactory fit between simulations and experimental data (see Section S6 for tabulated data of each ITIES studied). The dielectric constant of pure TFT is ∼9.18–9.47 at 30 °C and 18.3 °C, respectively,^82,83^ compared to ∼78.3 for water at 25 °C.^84^ The dielectric constant of a solution depends mainly on temperature, solvent composition, and electrolyte composition.^85^ In polar solvents, increasing electrolyte concentration typically lowers the static dielectric constant.^86^ In contrast, for organic solvents of moderate-to-low polarity, the dielectric constant may initially increase with electrolyte concentration (due to contact ion pair formation) before decreasing once solvation effects dominate.^87^ At higher electrolyte concentrations, more solvent molecules are bound within solvation shells, which limits solvent dipole reorientation and reduces the overall dielectric constant.^83^

The *ε*_TFT_ values used in our simulations reflect interfacial water solubility in TFT mediated by ions from the aqueous phase. For cells with LiCl (aqueous) and BATB (organic) electrolytes, *ε*_TFT_ ranged from 26 to 40, increasing with LiCl concentration (Table S5). This trend suggests that more solvated ions and associated water molecules partition into the organic phase as the LiCl concentration rises. In comparison, lower *ε*_TFT_ values between 14 and 23 were obtained for cells with Li_2_SO_4_ and BATB (Table S7). Since Li_2_SO_4_ is a kosmotropic salt and less soluble in TFT than LiCl, less water is transferred to the organic phase, yielding a smaller *ε*_TFT_. These findings align with the observations of Trojanek *et al.*, who linked water solubility in organic solvents to the Gibbs energy of cation transfer across ITIES prepared with 1,2-dichloroethane or TFT.^74^ They showed that cation transfer requires less energy when more water is dissolved in the organic phase. Likewise, our model supports this interpretation: trends in interfacial permittivity are closely tied to the extent of aqueous phase solubility within the organic phase.

### Non-applicability of Gouy–Chapman–Stern (GCS) theory to ITIES prepared with chemically reactive electrolytes

For electrolytes that undergo chemical reactions upon polarisation, GCS theory (two diffuse layers and an intermediate Helmholtz layer) cannot adequately describe either the measured differential capacitance profiles or the actual EDL structure. We observed these limitations at an ITIES formed with CaCl_2_ as the aqueous electrolyte (selected initially because of its 2 : 1 charge ratio). For a cell composed of 0.5 M CaCl_2_ and 5 mM BATB in TFT, CV and ACV cycling within a PPW defined by a ±2 µA cm^−2^ cutoff (CVs in Fig. 5A) led to the precipitation of a white solid at the three-phase junction (aqueous|TFT|glass, Fig. 5B). When the PPW cutoff was reduced to ± 1 µA cm^−2^, no visible crystallisation was observed, and the differential capacitance profiles in Fig. 5C were obtained. However, nanoscale crystallisation cannot be excluded. In fact, the differential capacitance measured at the highest CaCl_2_ concentration was lower than at more dilute concentrations (Fig. 5C(ii)), contrary to GCS predictions and clear evidence that localised CaCl_2_ precipitation had occurred.

Further inconsistencies emerged when varying the BATB concentration (Fig. 5C(i)). Simulations based on GCS theory and our three-capacitor series model yielded *ε*_TFT_ values equal to or even exceeding that of water (see Table S11), which is physically implausible. These unphysical dielectric constant outputs underscore the inapplicability of the model to such systems. In summary, the model developed here is valid only for ITIES prepared with aqueous and organic electrolytes that remain chemically inert under polarisation. For systems where polarisation triggers interfacial reactions, such as CaCl_2_-induced precipitation, GCS theory fails to represent either the capacitance behaviour or the interfacial structure.

## Conclusions

Electrochemistry at an ITIES remains an underexploited platform. Progress in this field depends on a rigorous interpretation of interfacial electrochemical processes (ion transfer, electron transfer, and adsorption), which requires a fundamental understanding of the electric double layer (EDL) at a polarisable liquid|liquid interface. In this work, we established an experimental methodology for obtaining accurate differential capacitance profiles at an ITIES and proposed a theoretical model of the EDL as an equivalent circuit of three capacitors in series (two diffuse layers and a Helmholtz layer, following Gouy–Chapman–Stern theory). An empirical equation was introduced to describe the dependence of the Helmholtz layer capacitance on the interfacial charge density. This model provided good agreement with the experimental results.

Using potentiodynamic electrochemical impedance spectroscopy (PDEIS), we identified the ideal polarisable potential window (PPW) for aqueous|TFT interfaces, where interfacial behaviour is fully capacitive. Extending beyond this window introduces non-capacitive, mass transport-driven processes. AC voltammetry (ACV) was also shown to modulate the balance between capacitive and faradaic ion transfer responses in the low-frequency regime (<100 Hz), with 80 Hz yielding profiles consistent with the proposed model. At higher frequencies (>100 Hz), impedance spectra revealed charge-transfer-like features sensitive to electrolyte concentration.

The fitted trends suggest that the Helmholtz layer capacitance depends on the aqueous electrolyte nature (including kosmotropic/chaotropic character and tendency of the ions to penetrate and accumulate in the mixed layer), the electrolyte concentrations on both sides of the interface, and the applied Δ^w^_o_*ϕ*. Additionally, the analysis indicated that the dielectric constant of the organic diffuse layer must be higher than that of bulk TFT, reflecting enhanced water content in the organic phase within the organic diffuse layer driven by ion solvation and transfer dynamics across the interface. The model is valid only for ITIES systems free from interfacial chemical reactions upon polarisation. Future work will focus on replacing the empirical expression for the Helmholtz layer capacitance with an analytical equation that captures its dependence on interfacial charge density.

## Author contributions

Juliana V. Díaz-Reyes: methodology, validation, formal analysis, investigation, data curation, writing – original draft, visualisation. Nicolás Rojas Sanabria: validation, investigation. Micheál D. Scanlon: conceptualisation, methodology, formal analysis, resources, data curation, writing – review & editing, visualisation, supervision, project administration, funding acquisition. Marco F. Suárez-Herrera: conceptualisation, methodology, formal analysis, resources, writing – original draft, visualisation, supervision, project administration.

## Conflicts of interest

There are no conflicts to declare.

## Supplementary Material

SC-OLF-D6SC03450K-s001

## Data Availability

All data are available in the main text or the supplementary information (SI). Additionally, the raw experimental and simulated data presented in this article, the Excel files used to model the differential capacitance profiles as a function of the interfacial Galvani potential difference, and a short movie detailing how to use the Excel files are available on Zenodo (https://doi.org/10.5281/zenodo.17808336). Supplementary information: theoretical description of the structure of the liquid|liquid interface; materials and methods; EIS and cyclic voltammetry data; differential capacitance modelling, parameter validation, fitting statistics, and capacitance contribution deconvolution for all ITIES studied. See DOI: https://doi.org/10.1039/d6sc03450k.
