# Supplementary material for: Dependence of the Helmholtz layer capacitance on interfacial charge density at polarisable liquid|liquid interfaces
Source: Chem Sci. 2026 Sep 24. Online ahead of print. doi: 10.1039/d6sc03450k (PMC13632635; doi:10.1039/d6sc03450k)
Supplement: Supplementary file 1 [file SC-OLF-D6SC03450K-s001.pdf]

Supplementary Information (SI) for Chemical Science.
This journal is © The Royal Society of Chemistry

Supplementary Information

Dependence of the Helmholtz Layer Capacitance on Interfacial Charge Density at Polarisable Liquid|Liquid Interfaces

Juliana V. Díaz-Reyes,[1] Nicolás Rojas Sanabria,[1] Micheál D. Scanlon,[1]* Marco F. Suárez-Herrera[2]*

[1]Department of Chemical Sciences and Bernal Institute, University of Limerick (UL), Limerick V94 T9PX, Ireland.

[2]Departamento de Química, Facultad de Ciencias, Universidad Nacional de Colombia, KR 30 No. 45-03, Edificio 451, Bogotá, Colombia.

*Corresponding author. Email: micheal.scanlon@ul.ie

*Corresponding author. Email: mfsuarezh@unal.edu.co

Table of Contents

Section	Content	Page
**S1**	Notes on the theoretical description of the structure of the liquid|liquid interface	S3
S1.1	Poisson-Boltzmann distributions for the diffuse layers	S3
S1.2	Demonstration of the analytical relationship between charge density and differential capacitance in either diffuse layer for symmetrically charged electrolytes	S3
S1.3	Differential capacitance of the Helmholtz layer	S4
S1.4	Fixed parameters used to compute the differential capacitance of polarisable aqueous|TFT interfaces	S5
S1.5	Summary of the Potential of Zero Charge (PZC) values for each of the electrolyte configurations studied	S6
**S2**	Materials and Methods	S7
S2.1	Reagents	S7
S2.2	Electrochemical measurements at a polarisable aqueous|TFT interface	S7
S2.3	Calibration of the electrochemical and modelled data to the Galvani scale and ($\Delta_o^w\phi - \Delta_o^w\phi_{PZC}$) scale	S9
S2.4	Electrochemical cell cleaning procedure	S9
S2.5	Assessing the purity of the chemicals and presence of contaminants in the four-electrode electrochemical cell when preparing an ITIES	S9
**S3**	EIS spectra recorded at the PZC for ITIES with LiCl, Li_2SO_4, MSA, or $CaCl_2$ aqueous electrolyte solutions and BATB organic electrolyte solutions	S12
S3.1	EIS spectra recorded at the PZC for the different ITIES systems studied	S12
S3.2	In-depth reasoning of why 80 Hz is chosen for the ACV measurements to generate differential capacitance profiles	S13
S3.3	Influence of aqueous and organic electrolyte concentrations on the EIS spectra at polarisable liquid|liquid interfaces	S14
S3.4	Effect of the instrument on the EIS spectra of an ITIES	S15
**S4**	Cyclic voltammetry for ITIES with aqueous electrolyte solutions of LiCl, Li_2SO_4, or MSA and organic electrolyte solutions of BATB	S16
S4.1	Effect of BATB concentration	S17
S4.2	Effect of aqueous electrolyte concentration	S17
**S5**	Parameter uniqueness in the calculation of differential capacitance at a polarisable liquid|liquid interface	S18
S5.1	Helmholtz capacitance functional form	S18
S5.2	Identifiability of the fitting parameters	S23
**S6**	Fitting variables and statistical goodness of fit analysis of the theoretical model to the measured differential capacitance curves for each ITIES studied	S26
**S7**	Deconvolution of the differential capacitance contributions for each of the ITIES modelled	S33
	Supporting references	S37

Section S1. Notes on the theoretical description of the structure of the liquid|liquid interface

S1.1 Poisson-Boltzmann distributions for the diffuse layers

The interfacial charge density is related to the potential drop of each diffuse layer, according to the following equations:

$$\sigma = \frac{q}{A} = \sqrt{2\varepsilon_r^{\mathrm{o}}\varepsilon_0 RT\left[c_+^{\mathrm{b,o}}\exp\left(-\frac{z_+^{\mathrm{o}}F\Delta\phi^{\mathrm{o}}(\sigma)}{RT}\right) + c_-^{\mathrm{b,o}}\exp\left(-\frac{z_-^{\mathrm{o}}F\Delta\phi^{\mathrm{o}}(\sigma)}{RT}\right) - \left(c_+^{\mathrm{b,o}} + c_-^{\mathrm{b,o}}\right)\right]} \quad \text{(S1)}$$

$$\sigma = \frac{q}{A} = \sqrt{2\varepsilon_r^{\mathrm{w}}\varepsilon_0 RT\left[c_+^{\mathrm{b,w}}\exp\left(\frac{z_+^{\mathrm{w}}F\Delta\phi^{\mathrm{w}}(\sigma)}{RT}\right) + c_-^{\mathrm{b,w}}\exp\left(\frac{z_-^{\mathrm{w}}F\Delta\phi^{\mathrm{w}}(\sigma)}{RT}\right) - \left(c_+^{\mathrm{b,w}} + c_-^{\mathrm{b,w}}\right)\right]} \quad \text{(S2)}$$

where $\Delta\phi(\sigma)$ is the potential drop function of the respective diffuse layer, c_+^{b} (c_-^{b}) and z_+ (z_-) are the bulk concentrations and charge values, respectively, of the cations (anions) in each phase, ε_r is the relative permittivity of the solvent where the respective diffuse layer is located, and the other terms have their usual meaning. Consequently, the differential capacitance of each diffuse layer is the derivative of the interfacial charge density with respect to the respective potential drop. Considering the electroneutrality condition of the bulk phases, $z_+c_+^{\mathrm{b,w}} + z_-c_-^{\mathrm{b,w}} = z_+c_+^{\mathrm{b,o}} + z_-c_-^{\mathrm{b,o}} = 0$, the differential capacitance of each diffuse layer as a function of potential is described by:

$$C_{\mathrm{d}}^{\mathrm{o}} = \frac{\mathrm{d}\sigma}{\mathrm{d}\Delta\phi^{\mathrm{o}}} = \frac{\varepsilon_{\mathrm{r}}^{\mathrm{o}}\varepsilon_0 F}{\sigma} z_+c_+^{\mathrm{b,o}}\left[\exp\left(-\frac{z_-^{\mathrm{o}}F\Delta\phi^{\mathrm{o}}}{RT}\right) - \exp\left(-\frac{z_+^{\mathrm{o}}F\Delta\phi^{\mathrm{o}}}{RT}\right)\right] \quad \text{(S3)}$$

$$C_{\mathrm{d}}^{\mathrm{w}} = \frac{\mathrm{d}\sigma}{\mathrm{d}\Delta\phi^{\mathrm{w}}} = \frac{\varepsilon_{\mathrm{r}}^{\mathrm{w}}\varepsilon_0 F}{\sigma} z_+c_+^{\mathrm{b,w}}\left[\exp\left(\frac{z_+^{\mathrm{w}}F\Delta\phi^{\mathrm{w}}}{RT}\right) - \exp\left(\frac{z_-^{\mathrm{w}}F\Delta\phi^{\mathrm{w}}}{RT}\right)\right] \quad \text{(S4)}$$

S1.2 Demonstration of the analytical relationship between charge density and differential capacitance in either diffuse layer for symmetrically charged electrolytes

The relationship between the charge density and the potential drop across the diffuse layers for any organic or aqueous electrolyte is described by equations (S1) and (S2). When an electrolyte has a symmetrical charge, the charge magnitudes of its anion and cation are equivalent ($z_+ = -z_- = z$), as are the concentrations in the bulk of each ionic species ($c_+^{\mathrm{b}} = c_-^{\mathrm{b}} = c^{\mathrm{b}}$). Note that although the magnitude of z for the anion and the cation is equivalent, its value is sign-sensitive,

resulting in each exponential having a different sign. Thus, equations (S1) and (S2) can be both simplified and rewritten as (S5):

$$\sigma = \sqrt{2\varepsilon_r\varepsilon_0 RTc^{\mathrm{b}}\left[\exp\left(-\frac{zF\Delta\phi(\sigma)}{RT}\right) + \exp\left(\frac{zF\Delta\phi(\sigma)}{RT}\right) - 2\right]} \tag{S5}$$

$$\sigma = \sqrt{4\varepsilon_r\varepsilon_0 RTc^{\mathrm{b}}\left[\cosh\left(\frac{zF\Delta\phi(\sigma)}{RT}\right) - 1\right]} \tag{S6}$$

Considering that $\cosh(2x) - 1 = 2\sinh^2(x)$:

$$\sigma = \sqrt{8\varepsilon_r\varepsilon_0 RTc^{\mathrm{b}}}\left[\sinh\left(\frac{zF\Delta\phi(\sigma)}{2RT}\right)\right] \tag{S7}$$

Equation (S7) is the usual solution for symmetrical electrolytes at electrode|electrolyte interfaces from Gouy-Chapman theory, also referred to as the Grahame equation. Consequently, the potential drop at each of the back-to-back diffuse layers can be calculated analytically from σ, as follows:

$$\Delta\phi(\sigma) = \left(\frac{2RT}{zF}\right)\left[\sinh^{-1}\left(\frac{\sigma}{\sqrt{8\varepsilon_r\varepsilon_0 RTc^{\mathrm{b}}}}\right)\right] \tag{S8}$$

Differentiating equation (S8), the differential capacitance of the corresponding diffuse layer (C_{d}) is:

$$C_{\mathrm{d}}(\sigma, \Delta\phi) = \frac{d\sigma}{d\Delta\phi(\sigma)} = \sqrt{\frac{2z^2F^2\varepsilon_r\varepsilon_0 c^{\mathrm{b}}}{RT}}\left[\cosh\left(\frac{zF\Delta\phi(\sigma)}{2RT}\right)\right] \tag{S9}$$

Note that the independent variable in equation (S9) is the potential drop calculated using equation (S8), which essentially represents the method of calculation proposed in the main text, where C_{d} is a nested function of the applied potential, and this in turn is a function of the interfacial charge density (σ), which is the independent variable of the entire system of equations.

S1.3 Differential capacitance of the Helmholtz layer

The differential capacitance of the Helmholtz layer was parameterised according to the piecewise function in equation (S10), to represent the asymmetry of the capacitance profile when the interfacial charge density (σ) is negative or positive.

$$C_{\mathrm{d}}^{\mathrm{H}}(\sigma) = \begin{cases} \alpha_1\, exp(-\beta_1|\sigma|) + \gamma_1, & \sigma \geq 0 \\ \alpha_2\, exp(-\beta_2|\sigma|) + \gamma_2, & \sigma < 0 \end{cases} \tag{S10}$$

where α_1, β_1, γ_1, α_2, β_2, and γ_2 are the empirical parameters.

The potential drop, $\Delta\phi^{\mathrm{H}}(\sigma)$, is then calculated from the definition of differential capacitance at the Helmholtz layer:

$$\Delta\phi^{\mathrm{H}}(\sigma) = \int_0^{\sigma} \frac{\mathrm{d}\sigma}{C_{\mathrm{d}}^{\mathrm{H}}(\sigma)} = \begin{cases} \dfrac{1}{\beta_1\gamma_1}\ln\dfrac{\alpha_1 + \gamma_1 \mathrm{e}^{\beta_1\sigma}}{\alpha_1 + \gamma_1}, & \sigma > 0 \\ 0, & \sigma = 0 \\ -\dfrac{1}{\beta_2\gamma_2}\ln\dfrac{\alpha_2 + \gamma_2 \mathrm{e}^{-\beta_2\sigma}}{\alpha_2 + \gamma_2}, & \sigma < 0 \end{cases} \tag{S11}$$

According to equation (S11), the parameters must follow the constraints: $\beta, \gamma \neq 0$, and $\alpha \neq -\gamma$.

S1.4 Fixed parameters used to compute the differential capacitance of polarisable aqueous|TFT interfaces

Table S1. Values of fundamental constants and fixed parameters for all electrochemical cell configurations investigated.

Category	Symbol	Value	Units
Fundamental constants*	R	8.314462618	J mol^{-1} K^{-1}
	F	96485.33212	C mol^{-1}
	ε_0	8.8541878188 ×10^{-12}	F m^{-1}
Fixed variables	T	293.15	K
	ε_r^w	78.304	
	z_{BA+}	1	
	z_{TB-}	-1	

*Taken from: NIST. CODATA Internationally recommended 2022 values of the Fundamental Physical Constants. Retrieved from: http://physics.nist.gov/constants

S1.5 Summary of the Potential of Zero Charge (PZC) values for each of the electrolyte configurations studied

Table S2. Summary of the Potential of Zero Charge (PZC) values on the $\Delta_o^w \phi$ scale, and the capacitance at the PZC, for three replicates of each of the electrolyte configurations studied.

**[Aq. electrolyte]** (mM)		**[BATB]** (mM)	$\Delta_o^w \phi_{PZC}$ (V)	C_{PZC} (μF cm^{-2})
[LiCl]	5.0	5.0	0.152 ± 0.005	5.30 ± 0.05
	10.0	5.0	0.139 ± 0.004	6.55 ± 0.46
	1000.0	5.0	0.125 ± 0.009	9.28 ± 0.57
	10.0	1.0	0.113 ± 0.007	3.45 ± 0.18
	10.0	10.0	0.1477 ± 0.0001	8.13 ± 0.07
[Li_2SO_4]	1.0	5.0	0.149 ± 0.003	4.19 ± 0.09
	5.0	5.0	0.131 ± 0.018	6.22 ± 0.29
	500.0	5.0	0.146 ± 0.008	7.24 ± 0.37
	5.0	1.0	0.115 ±0.006	3.04 ± 0.20
	5.0	10.0	0.156 ± 0.003	7.69 ± 0.27
[MSA]	15.3	5.1	0.147 ± 0.006	6.21 ± 0.52
	147.0	5.1	0.138 ± 0.011	6.11 ± 0.22
	1442.7	5.0	0.128 ± 0.008	7.37 ± 0.36
	15.3	1.1	0.123 ± 0.006	3.30 ± 0.18
	15.3	10.9	0.149 ± 0.008	8.47 ± 0.83
[$CaCl_2$]	1.0	5.0	0.145 ± 0.006	6.72 ± 0.49
	5.3	5.0	0.150 ± 0.013	8.43 ± 1.65
	500.3	5.0	0.134 ± 0.008	8.47 ± 2.20
	1.1	1.0	0.126 ± 0.007	3.33 ± 0.28
	1.0	10.0	0.147 ± 0.007	8.43 ± 0.53

Section S2. Materials and Methods

S2.1 Reagents

The chemicals were used as received without further purification unless specified below. All aqueous solutions were prepared with ultra-pure water (Millipore Milli-Q, specific resistivity 18.2 MΩ cm). Bis(triphenylphosphoranylidene)ammonium chloride (BACl, 97.0%) and lithium tetrakis(pentafluorophenyl)borate etherate (LiTB) were obtained from Sigma-Aldrich and Boulder Scientific Company, respectively. Bis(triphenylphosphoranylidene)ammonium tetrakis(pentafluorophenyl)borate (BATB) was prepared by metathesis of equimolar solutions of BACl and LiTB in a methanol-water 2:1 v/v mixture. The BATB salt was recrystallised in HPLC-grade ultrapure acetone (≥99.8%) from Fischer Scientific, until a reproducible cyclic voltammogram and differential capacitance curve were obtained for a "model" ITIES that is used as a reference system (see **Section S2.5** for more details). Lithium chloride (LiCl, ≥95%), tetraethylammonium chloride (TEACl, ≥98%), methanesulfonic acid (MSA, ≥99.0%), lithium sulfate (Li_2SO_4, ≥98.5%), sulfuric acid (H_2SO_4, ≥97.0%), and potassium permanganate ($KMnO_4$, ≥99.0%) were obtained from Sigma-Aldrich. Calcium chloride dried ($CaCl_2$, 97.0%) was purchased from Thermo Scientific and hydrogen peroxide (H_2O_2, ≥30.0%) from Honeywell, Fluka. All organic solutions were prepared in α,α,α-trifluorotoluene (TFT, also known as benzotrifluoride, 99.0%) from Thermo Scientific. As a pretreatment, the TFT was dried with molecular sieves 4Å from Sigma-Aldrich.

S2.2 Electrochemical measurements at a polarisable aqueous|TFT interface

All electrochemical measurements were conducted at polarisable ITIES formed using the four-electrode electrochemical cell configurations described in **Fig. 2A**. The custom-made four-electrode electrochemical cell, described previously,[1] was connected to an Autolab PGSTAT204 potentiostat equipped with a frequency response analyser module (FRA32M). Two reference electrodes positioned on either side of the liquid|liquid interface measured the interfacial Galvani potential difference ($\Delta_o^w \phi$). Platinum counter electrodes placed in each phase allowed the flow of electric current. The geometric surface area of the liquid|liquid interface was between 1.05–1.89 cm^2, depending on the glassware cell employed. The electrochemical cell was custom-made by Mulholland Scientific Glassblowing (Southampton, UK). The aqueous reference electrode was a commercial LowProfile Ag/AgCl KCl gel electrode from Pine Research (USA). The organic reference electrode was an Ag/AgCl wire

immersed in an organic reference solution (10.0 mM LiCl and 1.0 mM BACl in ultra-pure water) that established a liquid junction with the organic phase. All electrochemical measurements were performed inside a grounded Faraday cage, under ambient, aerobic conditions at room temperature.

For each ITIES investigated, the polarisable potential window (PPW) for potentiostatic Electrochemical Impedance Spectroscopy (EIS), Potentiodynamic Electrochemical Impedance Spectroscopy (PDEIS), and differential capacitance profiles obtained from Alternating Current voltammetry (ACV) measurements was established by cyclic voltammetry (CV) at 25 mV s^{-1} with current cutoffs of either ±1 or ±2 μA cm^{-2}. EIS and PDEIS spectra were measured across a frequency range of 0.1 Hz to 0.1 MHz, with a logarithmic distribution of 10 (EIS) or 15 (PDEIS) frequencies per decade, and an AC amplitude of 10 mV. EIS was measured at the potential of zero charge (PZC), while PDEIS was recorded every 0.05 V across the PPW. Differential capacitance profiles were obtained from ACV sweeps at a frequency of 80 Hz and 10 mV of amplitude. An uninterruptible power supply (APC, manufactured by Schneider Electric) was linked to the potentiostat to guarantee voltage stability and minimise inductive artefacts at high frequencies. For each four-electrode electrochemical cell configuration investigated, all replicates and the whole series of concentrations of aqueous and organic electrolytes were measured using the same electrochemical cell, connections, and potentiostat. Therefore, the impedance coming from instrumentation remained constant across all electrochemical measurements.

Under our experimental conditions, redox reactions at the counter electrodes in each phase are unlikely to generate detectable species, or “artifacts”, that could be sensed by charge transfer at the polarisable aqueous|TFT interface. Such detectable artifacts can be generated when the potentials at the counter electrodes become extreme, particularly in the organic phase. The individual potentials at each counter electrode can be monitored externally using a multimeter. For example, an applied $\Delta_{o}^{w}\phi$ of 0.4 V may correspond to a potential of +0.2 V at the aqueous counter electrode and –0.2 V at the organic counter electrode. These potentials increase when there is a large ion flux across the liquid|liquid interface. To compensate for this charge transfer, for instance, when anions move from the aqueous to the organic phase, the organic counter electrode must shift to more positive potentials. If the interfacial current becomes very high (e.g., ~100 μA cm^{-2}), redox reactions at the counter electrodes could generate products that might influence the chemistry at the ITIES. In our case, however, the

currents are low and the PPW is narrow (delimited by either ±1 or ±2 μA cm^{-2} cutoffs) to minimise ion transfer, and no ions are present in either phase that transfer within the PPW.

S2.3 Calibration of the electrochemical and modelled data to the Galvani scale and $(\Delta_o^w\phi - \Delta_o^w\phi_{PZC})$ scale

The cell potential (E) may be calibrated to the Galvani potential scale using the following relationship $E = \Delta_o^w\phi + \Delta E_{Ref}$. The critical value of ΔE_{Ref} was determined independently for each ITIES configuration studied using the electrochemical half-wave ion transfer response of tetraethylammonium cations (TEA^+; $E_{1/2,TEA^+}^{w\rightarrow TFT}$), and the standard ion transfer potential of TEA^+ from the aqueous to TFT phase (known to be $\Delta_o^w\phi_{tr,TEA^+}^{\ominus,w\rightarrow TFT} = 0.149$ V), as outlined previously.[2,3] From the measured differential capacitance curves, the value of $\Delta_o^w\phi_{PZC}$ was determined independently for each ITIES configuration studied (**Table S2**). The fittings of the differential capacitance curves were then carried out on the $(\Delta_o^w\phi - \Delta_o^w\phi_{PZC})$ scale, i.e., $\Delta_o^w\phi_{PZC}$ was set to 0 V for each ITIES configuration studied (as shown in **Fig. 3**).

S2.4 Electrochemical cell cleaning procedure

The four-electrode electrochemical cell was filled with permanganate solution (0.1 M $KMnO_4$ in 0.2 M H_2SO_4) overnight. After removing the permanganate solution, the cell was rinsed with piranha solution prepared using approximately 1 mL of H_2SO_4 (97% w/v) and 1 mL H_2O_2 (30% w/v) in 500 mL of water. Subsequently, the cell was rinsed with water to remove piranha residues; next, with boiling water three times, and then with acetone. Finally, the cell was dried in an oven at 80 °C and allowed to cool to room temperature before use. Further discussion on the observable characteristics in the cyclic voltammograms and differential capacitance profiles of a "model" ITIES that is used as a reference system to test if the electrochemical cell is clean or possibly contaminated is provided in the following section.

S2.5 Assessing the purity of the chemicals and presence of contaminants in the four-electrode electrochemical cell when preparing an ITIES

Electrochemical measurements at an ITIES are sensitive to the presence of contaminants in the biphasic system, particularly differential capacitance measurements. Several sources of

possible contamination can cause misleading results, for example, incomplete recrystallisation of the BATB organic electrolyte, impurities in the (as received) organic solvents, and contamination of the four-electrode electrochemical cell by species from previous experiments. From experience, we have found that ITIES prepared using the organic solvent 1,2-dichloroethane (DCE) do not present major contamination problems. However, when using the solvent TFT, the electrochemical measurements are far more susceptible to variation; thus, we recommend always drying the TFT with molecular sieves prior to use when preparing an ITIES.

As a diagnostic test to infer if contaminants are present in the biphasic system, we typically measure CVs and differential capacitance curves for a "reference biphasic system" consisting of 0.2 M H_2SO_4 as the aqueous electrolyte and 5 mM BATB in TFT as the organic electrolyte. If no contaminants are present, the potentiodynamic profiles shown in **Fig. S1** should be obtained. We suggest measuring it by setting a maximum current cutoff of ± 2.5 μA·cm^{-2}, as larger cutoffs (> ± 10 μA·cm^{-2}) may irreversibly disturb the liquid|liquid interface (for example by emulsifying the interface). The signals described in **Table S3** are typical if the biphasic system is contaminated.

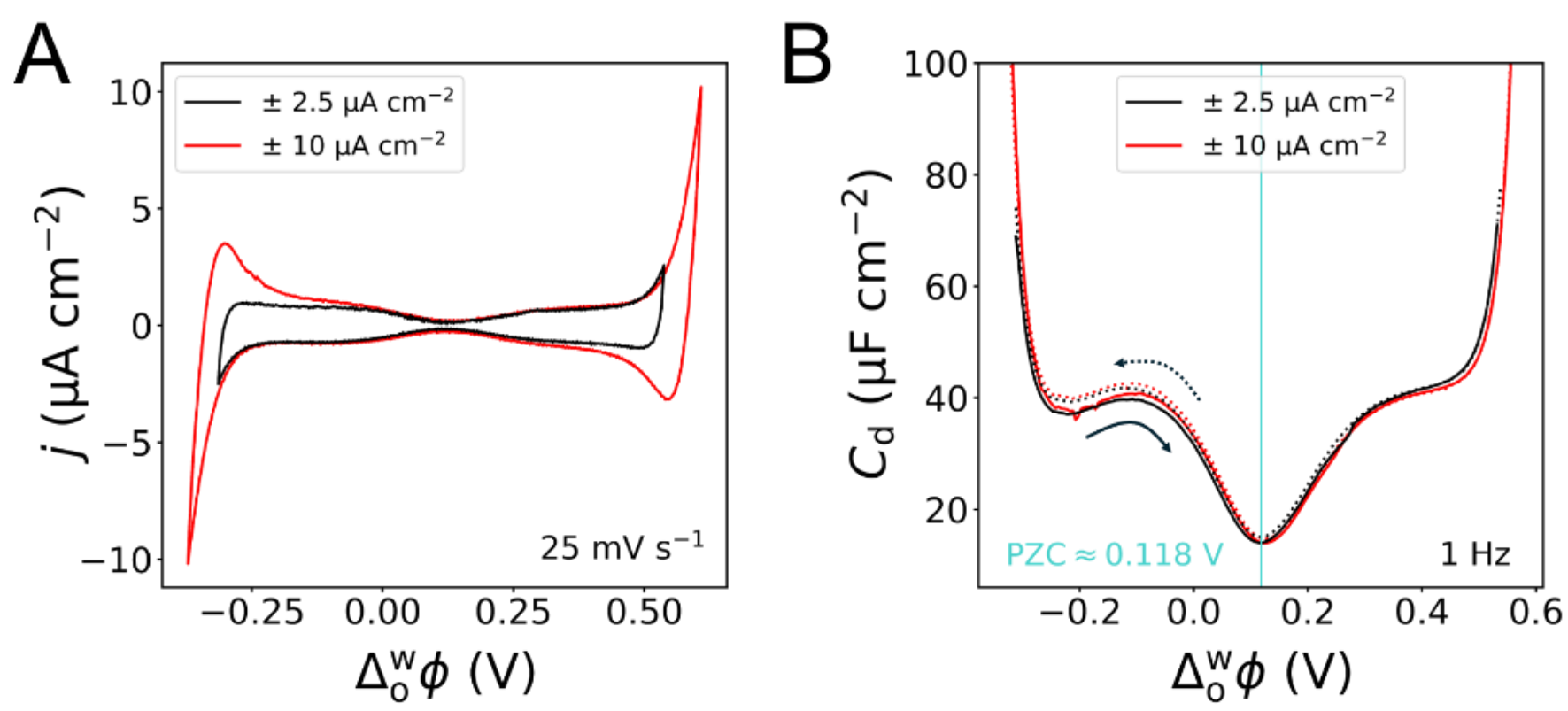

Fig. S1. Diagnostic test to infer if contaminants are present in the biphasic system for a "reference biphasic system". The latter consists of 0.2 M H_2SO_4 as the aqueous electrolyte and 5 mM BATB in TFT as the organic electrolyte. (**A**) Cyclic voltammograms recorded with the width of the PPW delimited by a current cutoff of either ± 2.5 or ± 10 μA·cm^{-2}. The scan rate used was 25 mV·s^{-1}. (**B**) Differential capacitance profiles obtained from ACV using the parameters described in the Section 3.2, with the PPW determined by the CVs in (A) with a current cutoff of either ± 2.5 or ± 10 μA·cm^{-2}. The arrows in (B) indicate the scan direction of the voltammogram.

Table S3. Observable characteristics in the CV and differential capacitance curve profiles when contamination is present in the four-electrode electrochemical cell.

Feature	CV profile	Differential capacitance profile
**Unexpected additional Faradaic events**	• Quasi-reversible signals.	• Peaks when measuring at low frequencies (1-5 Hz).
**PZC**	• Loss of symmetry with respect to the PZC.	• Loss of symmetry with respect to the PZC, and PZC shifting.
**Distortions**	• The shoulders formed at the beginning of the forward and backward sweeps are not defined and may appear broad. This can also occur at only one of the edges of the PPW.	• Irreproducible hysteresis: Significant differences when the ACV scan is obtained by scanning from positive to negative potentials, or *vice versa*.
**Capacitive current**	• The minimum capacitive currents in the centre of the PPW (located at the PZC) are not clearly defined.<br>• The distance of the capacitive currents from the forward to the backward sweep at around the PZC exceeds 1 $\mu A \cdot cm^{-2}$.	• Peaks when measuring at high frequencies (80-100 Hz).

Section S3. EIS spectra recorded at the PZC for ITIES with LiCl, Li_2SO_4, MSA, or $CaCl_2$ aqueous electrolyte solutions and BATB organic electrolyte solutions

S3.1 EIS spectra recorded at the PZC for the different ITIES systems studied

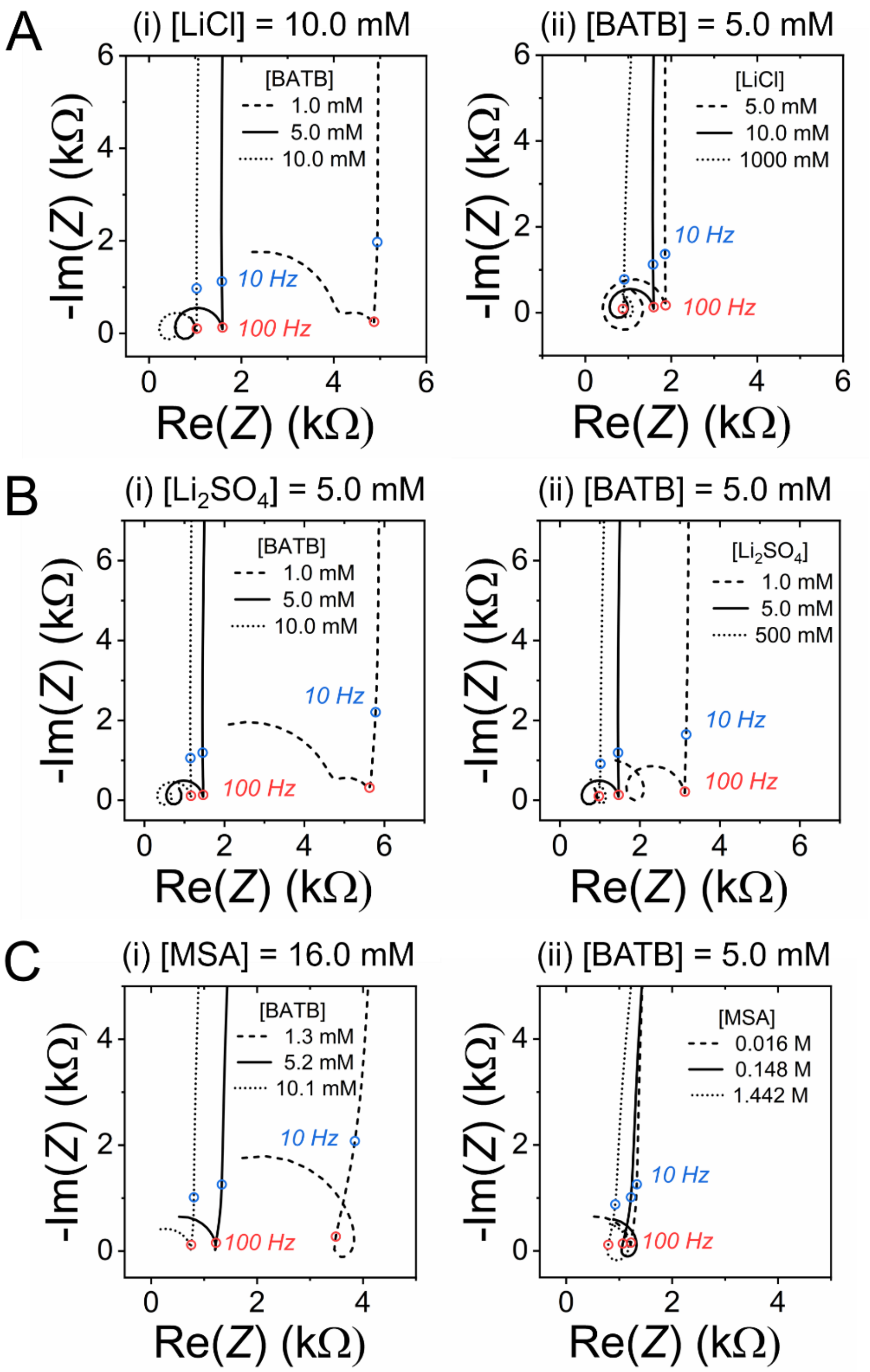

Fig. S2. Nyquist plots from EIS spectra recorded for ITIES prepared with aqueous electrolyte solutions of either (**A**) LiCl, (**B**) Li_2SO_4, or (**C**) MSA using the four-electrode electrochemical cell configuration described in Fig. 2A. The organic electrolyte was BATB in all cases. The concentrations of the various aqueous electrolytes and BATB are indicated in each panel. EIS

spectra were measured at the PZC ($\Delta_o^w\phi_{PZC}$ for each ITIES), across a frequency range of 0.1 Hz to 0.1 MHz with an AC amplitude of 10 mV. The labelled scattered points in each Nyquist plot show the location of the impedance point at 10 or 100 Hz, respectively. The complete set of EIS raw data is available on Zenodo (https://doi.org/10.5281/zenodo.17808336).

S3.2 In-depth reasoning of why 80 Hz is chosen for the ACV measurements to generate differential capacitance profiles

Before designing an ACV method to generate differential capacitance profiles, it is essential to understand how the system's impedance varies with the applied $\Delta_o^w\phi$. This ensures correct identification of the optimal PPW and measurement frequency (see main text). To illustrate why 80 Hz was chosen for the ACV measurements in this work, **Fig. S2** presents Nyquist plots recorded at $\Delta_o^w\phi_{PZC}$ for each ITIES studied. ACVs at polarisable liquid|liquid interfaces are usually measured in the 1–100 Hz range. In **Fig. S2**, two points in this range at 10 and 100 Hz are highlighted, while at 1 Hz, the imaginary impedance typically falls between –7 and –13 kΩ (the raw data files for the full spectrum or Bode plots are available on Zenodo https://doi.org/10.5281/zenodo.17808336). The impedance spectra show a marked change in behaviour around 100 Hz, separating the low- and high-frequency regions. In the low-frequency region, the real impedance remains nearly constant while the imaginary impedance increases as frequency decreases. This shape can be modelled by an ideal series RC circuit, where R represents the solution resistance and C the interfacial capacitance of the EDL. On these time scales (t > 0.01 s), the imaginary impedance primarily reflects EDL capacitance, making this frequency range suitable for differential capacitance measurements.

However, real systems often display *capacitance dispersion*, meaning the apparent capacitance depends on the applied frequency. At polarisable liquid|liquid interfaces, differential capacitance profiles exhibit higher magnitudes as the ACV measurement frequency decreases. For example, a 1 Hz profile typically shows higher capacitance than a 100 Hz profile, with the difference being almost constant across the PPW (as if one profile were translated relative to the other), suggesting *dielectric relaxation*. This occurs because dipoles and charges at the interface cannot instantly follow the applied AC electric field.[4] At lower frequencies, the dielectric has more time to respond to the potential disturbance, i.e., their relaxation time is shorter than the AC potential period, producing a higher apparent relative permittivity and capacitance. Furthermore, simulations using the model described in this work at 1 Hz require unreasonably high values of the dielectric constant of the TFT (ε_{TFT}) in the

organic diffuse layer (even exceeding that of bulk water), indicating that the capacitance is overestimated or that the theoretical model no longer applies at these timescales.

At very low frequencies (< 5 Hz), Faradaic processes also interfere. In Nyquist plots, this is seen as a mass-transport and/or charge-transfer "tail" because the real impedance is no longer constant and begins to increase, and the phase angle decreases, with decreasing frequency (as described for **Fig. 2, D and E** in the main text). These features become prominent when using acidic aqueous electrolytes or when polarising near the PPW limits. For instance, in the MSA system (**Fig. S2C)**, mass-transport contributions emerge below 2 Hz, especially at higher concentrations. To avoid such effects and emphasise capacitive behaviour, frequencies in the 80–100 Hz range are once more recommended.

S3.3 Influence of aqueous and organic electrolyte concentrations on the EIS spectra at polarisable liquid|liquid interfaces

Electrolyte concentration also influences the RC response. In the low-frequency region (< 100 Hz), solution resistance increases as electrolyte concentration decreases. This uncompensated resistance, in the context of EIS measurements, is a global property of the interface [5,6] and is not easily separated into aqueous and organic contributions. Nonetheless, the ohmic drop is more pronounced in the organic phase and can be quantified using closed-bipolar electrochemistry in a four-electrode configuration. The resistance of the solution at ITIES can be monitored by the real impedance in the low-frequency region, even when mass transport phenomena take place at the interface [7]. In this context, **Fig. S2** shows that increasing either aqueous or organic electrolyte concentration decreases solution resistance (i.e., increases conductivity). The effect is more pronounced for the organic electrolyte: resistance exceeds 4 kΩ at 1.0 mM BATB (**Fig. S2A(i) to C(i)**), compared to less than 3 kΩ for the most dilute aqueous electrolyte (**Fig. S2A(ii) to C(ii)**). This greater sensitivity of resistance to the organic electrolyte concentration highlights the dominant ohmic drop in the organic phase, as well manifested in the differential capacitance profiles discussed in the main text.

The high-frequency region (> 100 Hz) exhibits more complex behaviour, such as inductive loops and charge transfer–like semicircles. Although not the focus of this study, trends with electrolyte concentration are evident. Because all spectra were obtained using the same cell, electrodes, and instrumentation, variations among the profiles can be attributed to concentration effects. For example, inductive features disappear at 1.0 mM BATB in cells with

lithium salts (**Fig. S2A(i) and B(i)),** while they emerge in MSA-based cells (**Fig. S2C(i)).** Additionally, the size of these features tends to increase as the electrolyte concentration decreases, being more noticeable for concentration changes in organic electrolytes than in aqueous ones. It is possible that these loops are coupled to the resistance of the solution, and therefore, as the concentration decreases, their effect is magnified.

S3.4 Effect of the instrument on the EIS spectra of an ITIES

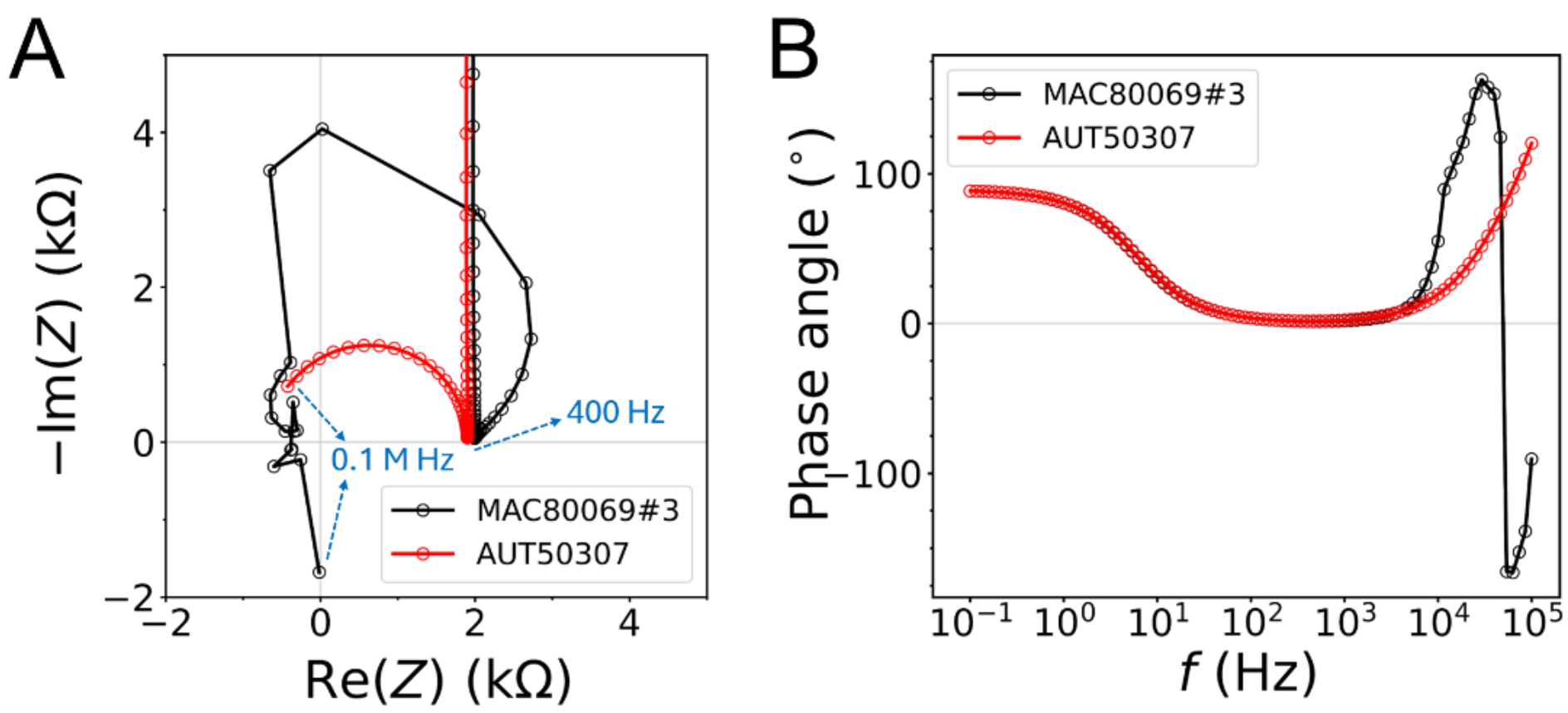

Fig. S3. EIS spectra were obtained using the cell configuration described in Fig. 2A with 1.0 mM $CaCl_2$ aqueous electrolyte and 10.0 mM BATB organic electrolyte (i.e., $x = 1.0$, $y = 10.0$, and $Z = \text{Cl}$). EIS spectra were measured at the PZC ($\Delta_o^w \phi_{PZC}$), across a frequency range of 0.1 Hz to 0.1 MHz with an AC amplitude of 10 mV. The (**A**) Nyquist and (**B**) phase angle Bode plots demonstrate the modification of the semicircle of high frequencies induced by measuring the spectrum with different potentiostats. Specifically, one measurement was made using an Autolab PGSTAT204 potentiostat (AUT50307) equipped with a frequency response analyser module (FRA32M) and the other with a Multi Autolab/M101 (MAC80069#3). The same four-electrode electrochemical cell and cell connections were used in both instances.

Section S4. Cyclic voltammetry for ITIES with aqueous electrolyte solutions of LiCl, Li_2SO_4, or MSA and organic electrolyte solutions of BATB

Fig. S4 depicts cyclic voltammograms of the ITIES configurations described in **Fig. 2A**, recorded at different aqueous and organic electrolyte concentrations. These CVs were obtained prior to any AC measurements in order to define the PPW for differential capacitance profiling. The PPW was delimited by applying an optimal current cutoff of ± 1 μA cm^{-2}, as justified in the main text. Comparison of the cyclic voltammograms across different systems reveals several electrochemical trends, discussed below.

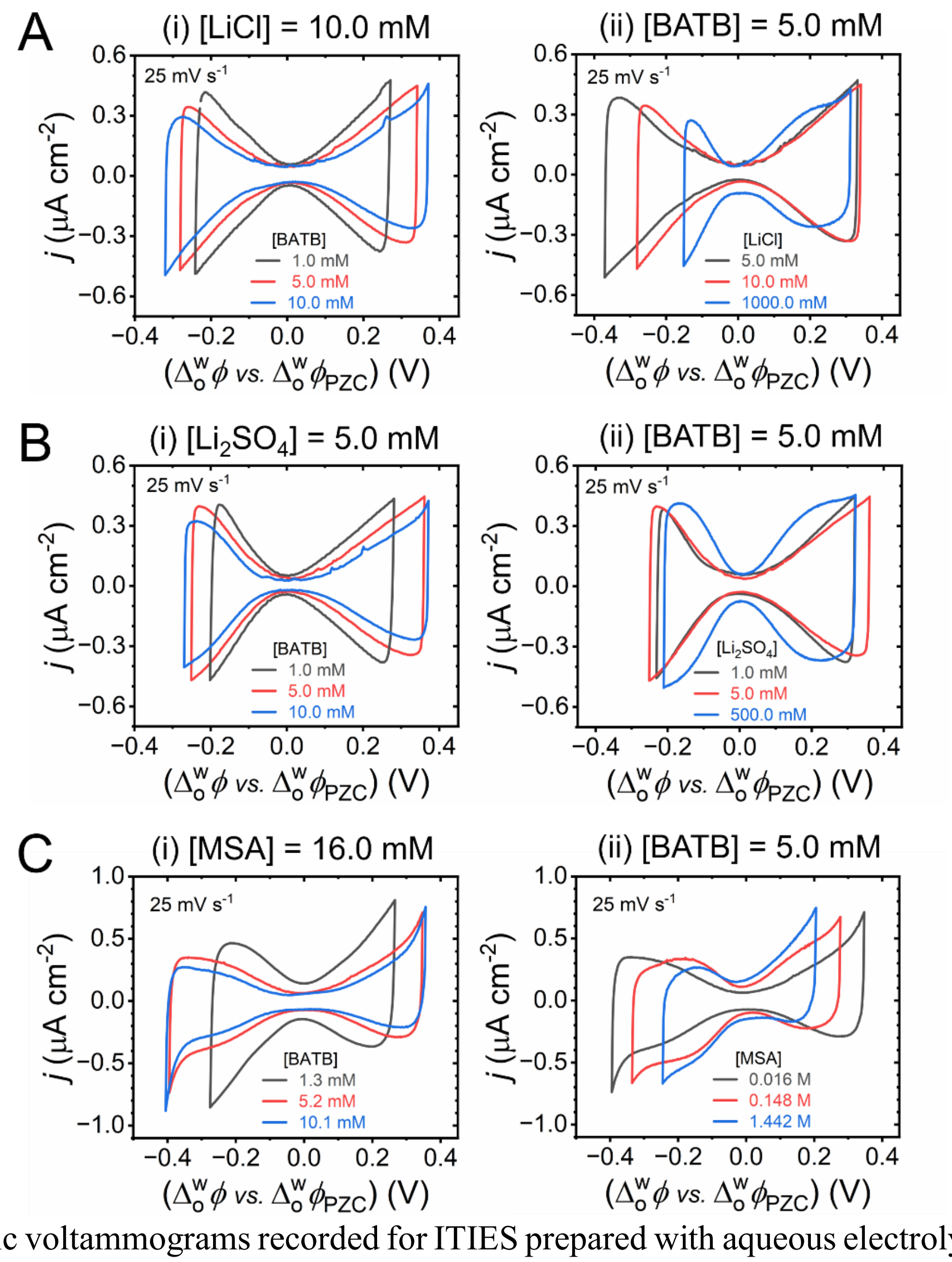

Fig. S4. Cyclic voltammograms recorded for ITIES prepared with aqueous electrolyte solutions of either (**A**) LiCl, (**B**) Li_2SO_4, or (**C**) MSA using the four-electrode electrochemical cell configuration described in Fig. 2A. The organic electrolyte was BATB in all cases. The concentrations of the various aqueous electrolytes and BATB organic electrolyte are indicated in each panel. The width of the PPW was delimited by a current cutoff of maximum ± 1 μA cm^{-2}.

S4.1 Effect of BATB concentration

Changing the concentration of BATB in the organic phase directly modulates the electrochemical processes that define the PPW. At liquid|liquid interfaces, the PPW limits are generally governed by ion transfer of electrolyte species, particularly from the aqueous phase: cations transfer under positive polarisation, while anions transfer under negative polarisation. According to the Nernst–Donnan relationship, increasing the concentration of the aqueous electrolyte lowers the potential required for ion transfer. Consistent with this, **Fig. S4A(ii) to C(ii)** show that the PPW narrows as the aqueous electrolyte concentration increases. However, **Fig. S4A(i) to C(i)** demonstrate that the PPW also narrows with decreasing BATB concentration in the organic phase. This effect cannot be attributed solely to ion transfer: impedance spectra from **Fig. S3** shows that solution resistance rises markedly at lower BATB concentrations, indicating that resistive contributions may also shorten the PPW.

S4.2 Effect of aqueous electrolyte concentration

At higher aqueous electrolyte concentrations (≥ 0.5 M), capacitive currents within the PPW become more pronounced, as reflected in the curvature of the blue solid line profiles in **Fig. S4A(ii) and B(ii)**. At these concentrations, Li^+ tends to accumulate at the interface. For equivalent bulk Li^+ concentrations (1 M), Li_2SO_4 produces earlier and larger accumulation currents than LiCl. This behaviour aligns with ion-specific effects: both Li^+ and SO_4^{2-} are kosmotropic, favouring interfacial stability by reinforcing water structure. In contrast, Cl^- is chaotropic, disrupting water structure and making it more susceptible to ion transfer, which explains the shorter PPW observed as its concentration increases.

Section S5. Parameter uniqueness in the calculation of differential capacitance at a polarisable liquid|liquid interface

S5.1. Helmholtz capacitance functional form

Recapping the main text, the Helmholtz capacitance (C_d^H) of a polarisable liquid|liquid interface must account for:

i. A "Discrete Helmholtz" behaviour when the ionic electrolyte concentration builds up at the interface, due to polarisation or increased electrolyte bulk concentration. At increased polarisation, the total equivalent capacitance is essentially given by C_d^H, which remains constant due to electrostatic repulsion and the finite size of the accumulated ions, and decreases relative to its value at the PZC due to solvent polarisation (dielectric saturation).
ii. A negligible contribution of C_d^H to the total equivalent capacitance at the PZC, where the diffuse layers predominate and the Helmholtz layer is practically non-existent.
iii. Variation of C_d^H with the polarisation applied to the interface, whether as a function of interfacial charge density, $\Delta_\mathrm{o}^\mathrm{w}\phi$, or electric field.
iv. Asymmetry of C_d^H due to the ability of the liquid|liquid interface to separate cationic and anionic contributions from the organic and the aqueous side by polarisation.
v. C_d^H may be on the order of hundreds of μF cm^{-2}. For example, C_d^H can reach 350-400 μF cm^{-2} for aqueous|nitrobenzene interfaces, as shown by Samec and co-workers.[8,9]

For these reasons, a peak-shaped (or bell-shaped) asymmetrical function centred at the PZC is a reasonable profile form to represent C_d^H as a function of the interfacial charge density. However, various mathematical formulations can resemble this behaviour. Therefore, to justify the semiempirical form of equation (5) in the main text, we compared the Bayesian Information Criterion (BIC) values obtained from fitting a liquid|liquid electrochemical cell to different mathematical functions that can account for the features listed above.

In summary, BIC is a statistical metric used to compare models and score them based on model accuracy and simplicity, with a strong penalty for overfitting, for example, due to an increased number of free parameters. In this test, a model with a smaller BIC is preferred. It is calculated from the log-likelihood, according to:

$$\ell = \ln(\mathcal{L}) = -\frac{N}{2}\ln(2\pi\sigma^2) - \frac{1}{2}\sum_{i}^{N}\frac{\left(C_{d,i} - \hat{C}_{d,i}\right)^2}{\sigma^2} \tag{S12}$$

$$\mathrm{BIC} = k\ln(N) - 2\ln(\mathcal{L}) \tag{S13}$$

where ℓ is the maximised log-likelihood, N is the number of data points measured, σ^2 is the variance, $\left(C_{d,i} - \hat{C}_{d,i}\right)$ are the residuals, and k is the number of free parameters to fit in the model. The maximised log-likelihood and BIC scores for all the simulations presented in the main text are reported in the goodness-of-fit section of the corresponding Excel files in Zenodo (https://doi.org/10.5281/zenodo.17808336).

Another point to consider when proposing a capacitance model for the Helmholtz layer is the calculation of the potential difference; for this, the definition of the differential capacitance is employed, and the potential at the Helmholtz layer ($\Delta\phi^{H}$) is found by solving the integral:

$$\Delta\phi^{H}(\sigma) = \int_0^{\sigma}\frac{d\sigma}{C_d^{H}(\sigma)} \tag{S14}$$

Some capacitance and potential equations assessed are listed from least to most complex, and their BIC scores are reported in **Table S4**. The models were parameterised symmetrically and asymmetrically with respect to the sign of the interfacial charge density (σ). In all cases, asymmetry improves the fit considerably, despite involving the inclusion of more parameters (see BIC values in **Table S4**).

The Gouy-Chapman-Stern (GCS) approach was first implemented, where C_d^{H} is constant with respect to σ (**Fig. S5A**), as discussed in the main text, this results in parabolic equivalent capacitances that tend to infinity as the charge density increases. Being the simplest model, it completely masks the plateaus associated with processes at the Helmholtz layer. The specific value of 40 μA cm^{-2} was selected for illustrative purposes. It is a realistic capacitance value and closely matches the data from the cell configuration using LiCl as the aqueous electrolyte presented in **Fig. 3**. Technically, values between 40 and 50 μF cm^{-2} may provide the best correlation with these data. **Fig. S6** shows the variation of the equivalent capacitance profile with the Helmholtz capacitance constant. This graph was constructed using a realistic range of Helmholtz capacitance values from 10 to 100 μF cm^{-2}. It is evident that when C_d^{H} is held constant, the profile shape from the GCS model does not fully reproduce all the characteristics of the experimental data.

To model the plateaus associated with processes at the Helmholtz layer, the variation of $C_{\mathrm{d}}^{\mathrm{H}}$ with respect to σ was introduced. The absolute value and quadratic terms produce peak- and bell-shaped profiles with respect to σ. Similarly, linear (**Fig. S5B**), parabolic (**Fig. S5C**), and exponential (**Fig. S5D to F**) functions were evaluated. Within these functional forms, absolute value and exponentials yield the best correlation with the data and very low BIC scores.

The free parameters to fit include the semiempirical constants to calculate $C_{\mathrm{d}}^{\mathrm{H}}$ and $\Delta\phi^{\mathrm{H}}$, i.e. α, β, and γ; and the dielectric constant of the organic diffuse layer, $\varepsilon_{\mathrm{TFT}}$. In addition to the goodness-of-fit statistics, the $\varepsilon_{\mathrm{TFT}}$ estimate indicates the quality of the semiempirical functional form employed in the fit. When the semi-empirical part is oversimplified, the value of $\varepsilon_{\mathrm{TFT}}$ is overestimated ($\varepsilon_{\mathrm{TFT}} > 50$), since the simulation of the equivalent capacitance profile fails to adequately correlate the points around the PZC, that is, the region mainly influenced by the differential capacitance of the diffuse layers.

Among the semiempirical models studied, the best fit was achieved by employing a combination of exponentials in an asymmetrical configuration, which is a Butler-Volmer-like equation; however, this approach involves 11 free parameters, and the integral in equation () is not defined for this model, which involves an additional numerical calculation that increases the fitting error. For this reason, equation (5) was preferred to model $C_{\mathrm{d}}^{\mathrm{H}}$, since it has an analytical solution for the potential, it doesn't overestimate $\varepsilon_{\mathrm{TFT}}$, and yields a high correlation with the fewest possible free parameters.

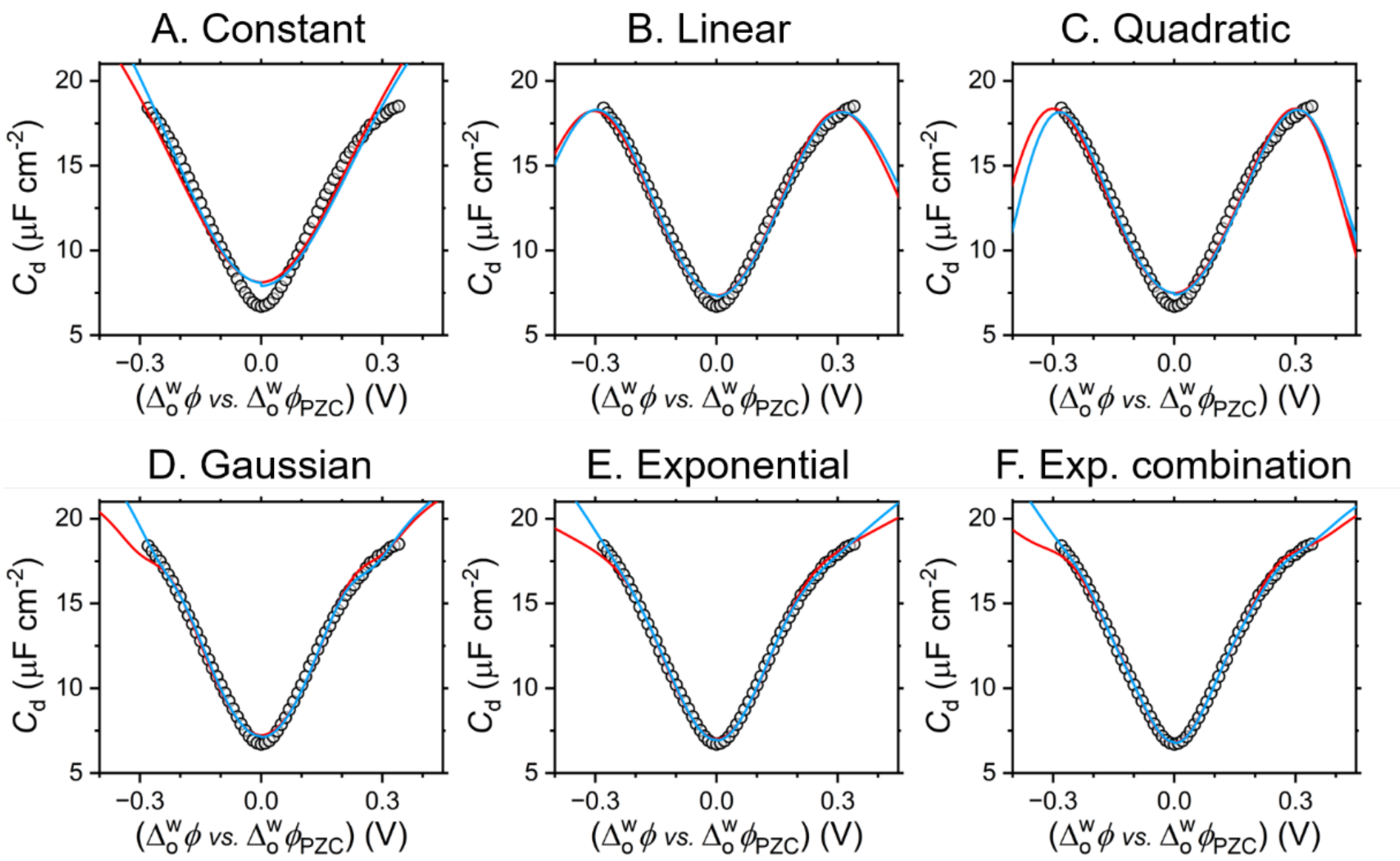

Fig. S5. Simulation of the differential capacitance profile recorded for an ITIES prepared with an aqueous electrolyte solution of 10.0 mM LiCl and organic electrolyte solution of 5.0 mM BATB in TFT using the four-electrode electrochemical cell configuration described in Fig. 2A. Different semiempirical equations, **(A)** Constant, **(B)** Linear, **(C)** Quadratic, **(D)** Gaussian, **(E)** Exponential, and **(F)** Exponential Combination, were used to calculate the Helmholtz layer differential (C_d^H). Profiles of the equivalent differential capacitance as a function of the Galvani potential normalised to the PZC. Symmetrical (red line) and asymmetrical (blue line) equations versus the interfacial charge density.

Table S4. Possible semiempirical models of differential capacitance (C_d^H) and potential difference ($\Delta\phi^H$) of the Helmholtz layer at a polarisable liquid|liquid interface. Comparison between symmetric and asymmetric formulations relative to the interfacial charge density (σ), dielectric constant of the organic diffuse layer (ε_{TFT}), total number of free parameters (k) and BIC for the simulation of the cell: LiCl 10.0 mM | BATB 5.0 mM, TFT.

Semiempirical Model	Helmholtz differential capacitance	Helmholtz potential difference	$\varepsilon_{\mathrm{TFT}}$	k	BIC
Constant Symmetrical	$C_\mathrm{d}^\mathrm{H} = \gamma$	$\Delta\phi^\mathrm{H} = \frac{\sigma}{C_d^H} = \frac{\sigma}{\gamma}$	105.8	2	171
Constant Asymmetrical	$C_\mathrm{d}^\mathrm{H}(\sigma) = \begin{cases} \gamma_1, & \sigma \geq 0 \\ \gamma_2, & \sigma < 0 \end{cases}$	$\Delta\phi^\mathrm{H}(\sigma) = \begin{cases} \frac{\sigma}{\gamma_1}, & \sigma \geq 0 \\ \frac{\sigma}{\gamma_2}, & \sigma < 0 \end{cases}$	95.9	3	158
Linear Symmetrical	$C_\mathrm{d}^\mathrm{H}(\sigma) = -\beta\lvert\sigma\rvert + \gamma$	$\Delta\phi^\mathrm{H}(\sigma) = \begin{cases} \frac{1}{\beta}\ln\left(\frac{\gamma}{\gamma - \beta\sigma}\right), & \sigma \geq 0 \\ \frac{1}{\beta}\ln\left(\frac{\gamma + \beta\sigma}{\gamma}\right), & \sigma < 0 \end{cases}$	50.4	3	57
Linear Asymmetrical	$C_\mathrm{d}^\mathrm{H}(\sigma) = \begin{cases} -\beta_1\lvert\sigma\rvert + \gamma_1, & \sigma \geq 0 \\ -\beta_2\lvert\sigma\rvert + \gamma_2, & \sigma < 0 \end{cases}$	$\Delta\phi^\mathrm{H}(\sigma) = \begin{cases} \frac{1}{\beta_1}\ln\left(\frac{\gamma_1}{\gamma_1 - \beta_1\sigma}\right), & \sigma \geq 0 \\ \frac{1}{\beta_2}\ln\left(\frac{\gamma_2 + \beta_2\sigma}{\gamma_2}\right), & \sigma < 0 \end{cases}$	50.1	5	53
Quadratic Symmetrical	$C_\mathrm{d}^\mathrm{H}(\sigma) = -\beta\sigma^2 + \gamma$	$\Delta\phi^\mathrm{H} = \sqrt{\frac{1}{\beta\gamma}}\mathrm{artanh}\left(\sqrt{\frac{\beta}{\gamma}}\sigma\right)$	60.2	3	83
Quadratic Asymmetrical	$C_\mathrm{d}^\mathrm{H}(\sigma) = \begin{cases} -\beta_1\sigma^2 + \gamma_1, & \sigma \geq 0 \\ -\beta_2\sigma^2 + \gamma_2, & \sigma < 0 \end{cases}$	$\Delta\phi^\mathrm{H}(\sigma) = \begin{cases} \sqrt{\frac{1}{\beta_1\gamma_1}}\mathrm{artanh}\left(\sqrt{\frac{\beta_1}{\gamma_1}}\sigma\right), & \sigma \geq 0 \\ \sqrt{\frac{1}{\beta_2\gamma_2}}\mathrm{artanh}\left(\sqrt{\frac{\beta_2}{\gamma_2}}\sigma\right), & \sigma < 0 \end{cases}$	58.5	5	80
Gaussian Symmetrical	$C_\mathrm{d}^\mathrm{H} = \alpha\, e^{-\beta\sigma^2} + \gamma$	$\Delta\phi^\mathrm{H}(\sigma) = \int_0^\sigma \frac{\mathrm{d}\sigma}{C_\mathrm{d}^\mathrm{H}(\sigma)}$ Not defined, a numerical calculation must be used.	65.6	4	49
Gaussian Asymmetrical	$C_\mathrm{d}^\mathrm{H}(\sigma) = \begin{cases} \alpha_1 e^{-\beta_1\sigma^2} + \gamma_1, & \sigma \geq 0 \\ \alpha_2 e^{-\beta_2\sigma^2} + \gamma_2, & \sigma < 0 \end{cases}$	$\Delta\phi^\mathrm{H}(\sigma) = \int_0^\sigma \frac{\mathrm{d}\sigma}{C_\mathrm{d}^\mathrm{H}(\sigma)}$ Not defined, a numerical calculation must be used.	56.7	7	10
Exponential Symmetrical	$C_\mathrm{d}^\mathrm{H} = \alpha e^{-\beta\lvert\sigma\rvert} + \gamma$	$\Delta\phi^\mathrm{H}(\sigma) = \begin{cases} \frac{1}{\beta\gamma}\ln\left(\frac{\alpha + \gamma \mathrm{e}^{\beta\sigma}}{\alpha + \gamma}\right), & \sigma \geq 0 \\ -\frac{1}{\beta\gamma}\ln\left(\frac{\alpha + \gamma \mathrm{e}^{-\beta\sigma}}{\alpha + \gamma}\right), & \sigma < 0 \end{cases}$	35.8	4	9
Exponential Asymmetrical (This work)	$C_\mathrm{d}^\mathrm{H}(\sigma) = \begin{cases} \alpha_1 e^{-\beta_1\lvert\sigma\rvert} + \gamma_1, & \sigma \geq 0 \\ \alpha_2 e^{-\beta_2\lvert\sigma\rvert} + \gamma_2, & \sigma < 0 \end{cases}$	$\Delta\phi^\mathrm{H}(\sigma) = \begin{cases} \frac{1}{\beta_1\gamma_1}\ln\left(\frac{\alpha_1 + \gamma_1 \mathrm{e}^{\beta_1\sigma}}{\alpha_1 + \gamma_1}\right), & \sigma \geq 0 \\ -\frac{1}{\beta_2\gamma_2}\ln\left(\frac{\alpha_2 + \gamma_2 \mathrm{e}^{-\beta_2\sigma}}{\alpha_2 + \gamma_2}\right), & \sigma < 0 \end{cases}$	33.2	7	-66

Exponential combination Symmetrical	$C_{\mathrm{d}}^{\mathrm{H}}(\sigma) = \alpha_1 e^{-\beta_1\lvert\sigma\rvert} + \alpha_2 e^{-\beta_2\lvert\sigma\rvert} + \gamma$	$\Delta\phi^{\mathrm{H}}(\sigma) = \int_0^{\sigma} \frac{\mathrm{d}\sigma}{C_{\mathrm{d}}^{\mathrm{H}}(\sigma)}$ Not defined, a numerical calculation must be used.	28.8	6	-5
Exponential combination Asymmetrical	$C_{\mathrm{d}}^{\mathrm{H}}(\sigma) = \begin{cases} \alpha_1 e^{-\beta_1\lvert\sigma\rvert} + \alpha_2 e^{-\beta_2\lvert\sigma\rvert} + \gamma_1, & \sigma \geq 0 \\ \alpha_3 e^{-\beta_3\lvert\sigma\rvert} + \alpha_4 e^{-\beta_4\lvert\sigma\rvert} + \gamma_2, & \sigma < 0 \end{cases}$	$\Delta\phi^{\mathrm{H}}(\sigma) = \int_0^{\sigma} \frac{\mathrm{d}\sigma}{C_{\mathrm{d}}^{\mathrm{H}}(\sigma)}$ Not defined, a numerical calculation must be used.	28.6	11	-147

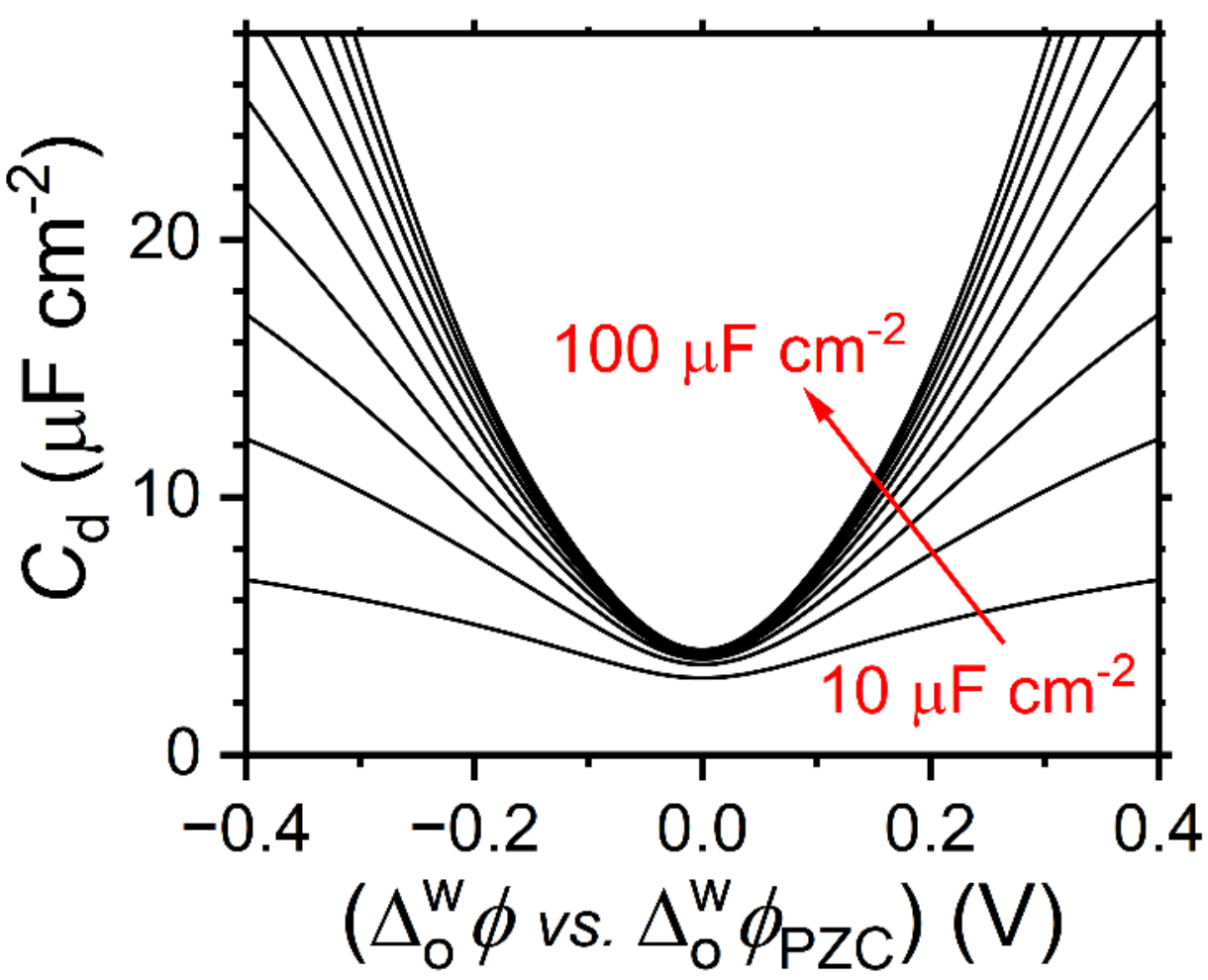

Fig. S6. Effect of the variation of $C_{\mathrm{d}}^{\mathrm{H}}$ as a constant (GCS model) in the calculated equivalent capacitance profile for an ITIES prepare with 10 mM aqueous electrolyte (charge-symmetrical) and organic electrolyte solution of 5 mM BATB in TFT.

S5.2. Identifiability of the fitting parameters

To further assess the uniqueness of the fitted parameters, a profile likelihood analysis of the model proposed in the main text was performed. The log-likelihood was used as the objective function to be maximised (equation (S12)). The difference relative to the global optimum for each ITIES studied was then computed to determine confidence intervals for each parameter using the profile-likelihood test (equation ()).

$$\Delta(2\ell)_p = 2(\ell_{global} - \ell_p) \tag{S15}$$

where ℓ_p is the maximised log-likelihood with parameter p held fixed, and ℓ_{global} is the maximised log-likelihood of the global optimal solution.

A dataset for ITIES prepared with an aqueous electrolyte solution of 16.0 mM MSA and organic electrolyte solutions of varying BATB concentration in TFT was chosen as the analysis sample, given the greater sensitivity of the capacitance at a polarisable liquid|liquid

interface to the organic electrolyte concentration. **Fig. S7** shows the profile likelihood values of the fitting parameters for the chosen dataset. In all cases, it was found that the parameters α_1, α_2, β_1, β_2, γ_1, γ_2, and ε_{TFT} are structurally and practically identifiable; each has a finite-size confidence interval, as their profiles exhibit well-defined minima and finite two-sided $\Delta(2\ell)$ bounds crossing the 95% threshold.

Confidence intervals were derived using the likelihood ratio test, with thresholds based on the χ^2 distribution, i.e. $\Delta(-2\log\mathcal{L})$ = 3.84 for 95% confidence with one degree of freedom. Confidence intervals indicate the range within which the data account for the parameters, and this range is finite and specific to each ITIES configuration. The range of values over which the same parameter (for example, α_1) for a set of ITIES with varying electrolyte concentrations can fit is of the same order of magnitude, i.e., it is not completely random and reflects trends within the set. Even if the fitting strategy involves a free variation of the parameters by a trial-and-error initial guess, the best-fit parameters and their 95% confidence intervals are specific and constrained. This is due to the use of the minimisation of RMSE and χ^2 as optimisation criteria, which implies maximum likelihood; therefore, only a specific combination of parameter values can yield the optimum, as visually evidenced by the minima in the profile likelihoods in **Fig. S7**.

Another feature from this analysis is the widening of the confidence interval when a very dilute organic electrolyte solution is tested ([BATB] ≈ 1 mM). The latter demonstrates that practical identifiability improves with concentration, and data is information-limited at low concentrations. This does not necessarily reveal inconsistencies with the proposed mathematical formulation; rather, it shows how the estimate of C_d^H changes when the electrolyte concentration is significantly reduced, especially on the organic side. When the electrolyte concentration is very low, the Helmholtz layer is practically negligible, and thus the data are less informative about the C_d^H contribution.

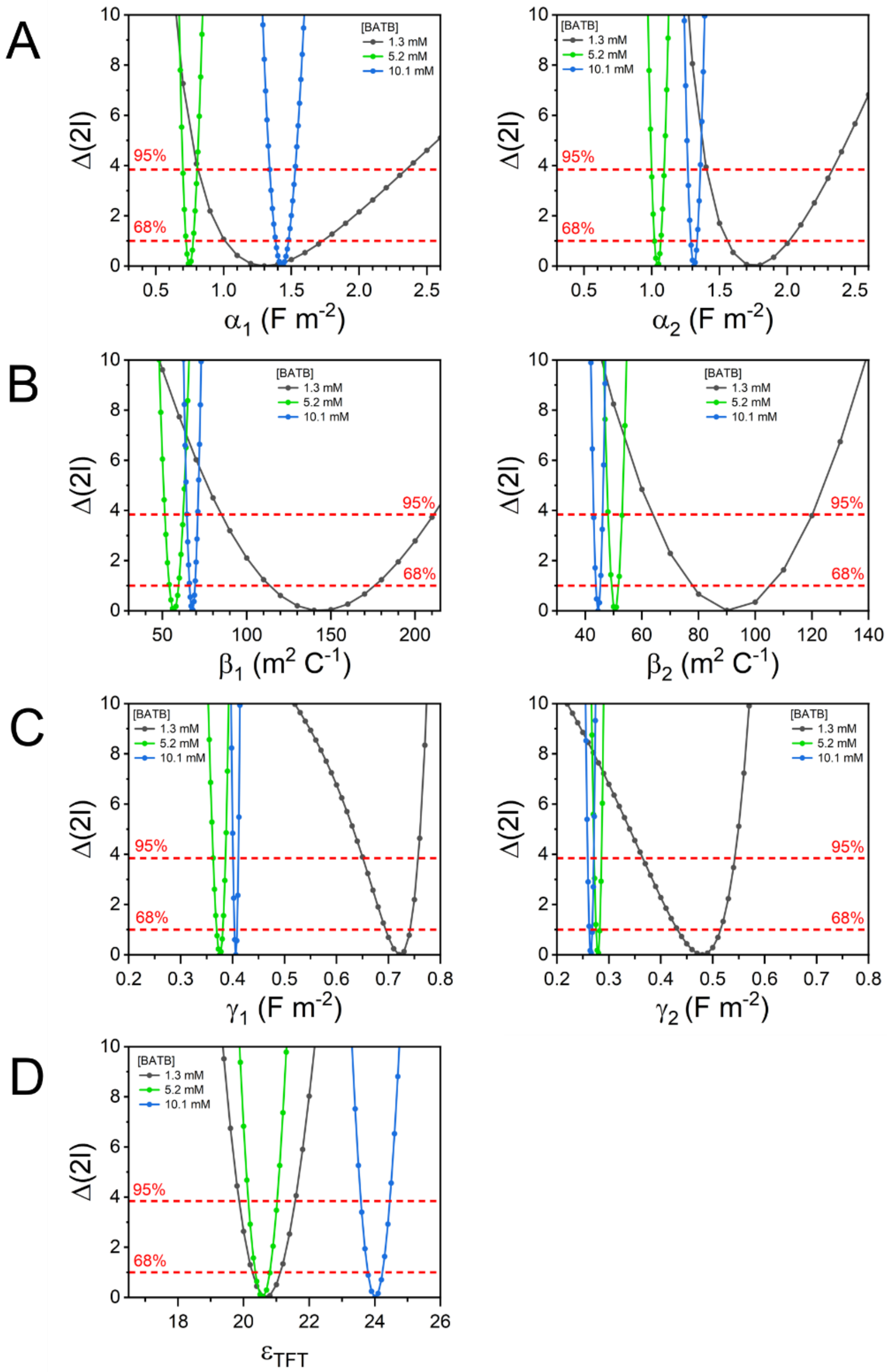

Fig. S7. Profile likelihood of the Helmholtz capacitance parameters **(A)** α, **(B)** β, **(C)** γ, and **(D)** ε_{TFT}, calculated for an ITIES prepared with an aqueous electrolyte solution of 16.0 mM MSA and various concentrations (*x* mM) of BATB in TFT. The red dotted lines indicate the 95% and 68% confidence interval thresholds.

Section S6. Fitting variables and statistical goodness of fit analysis of the theoretical model to the measured differential capacitance curves for each ITIES studied

The profiles shown in the main text (**Fig. 3**) were modelled in Microsoft Excel (these Microsoft Excel files are available for download on Zenodo: https://doi.org/10.5281/zenodo.17808336). The vectors characterising the differential capacitance of the polarisable liquid|liquid interface were calculated based on the GCS theory, including a semiempirical description of the Helmholtz layer. These theoretical profiles were then compared with the experimental ACV-derived profiles, plotted on the $(\Delta_o^w\phi - \Delta_o^w\phi_{PZC})$ scale. The fitting parameters were: the relative permittivity of the organic diffuse layer (ε_{TFT}); the semi-empirical parameters of the differential capacitance function of the Helmholtz layer ($\alpha_1, \beta_1, \gamma_1, \alpha_2, \beta_2$, and γ_2; see equation (S10)); and the offset in the PZC (ΔPZC), only in specific cases, as explained *vide infra*. Fitting was first optimised manually by trial and error until good visual agreement was achieved. Residuals were evaluated to ensure randomness, and the fit was refined using Solver to minimise the Root-Mean-Square Error (RMSE). The final optimised parameters are reported in the following tables.

The value of the relative permittivity of the aqueous phase (ε_r^w) was fixed as 78.304 (see **Table S1**) to reduce the number of parameters to be fitted, since reasonable changes in ε_r^w (between 40 and 100) do not significantly affect the modelled profile (**Fig. S8**). It is worth noting that at dilute concentrations of aqueous electrolytes, this parameter can be slightly higher than the dielectric constant of pure water, whereas at high electrolyte concentrations it may decrease (a phenomenon known as dielectric decrement; see, for instance, Fig. 1 in supporting reference [10]). However, as noted in the main text when discussing the data presented in **Fig. 3**, the differential capacitance was more sensitive to changes in organic electrolyte concentration than to changes in aqueous electrolyte concentration. This is due to the greater resistance of the organic solution; thus, ε_r^w was fixed and changes in ε_{TFT} were solely analysed.

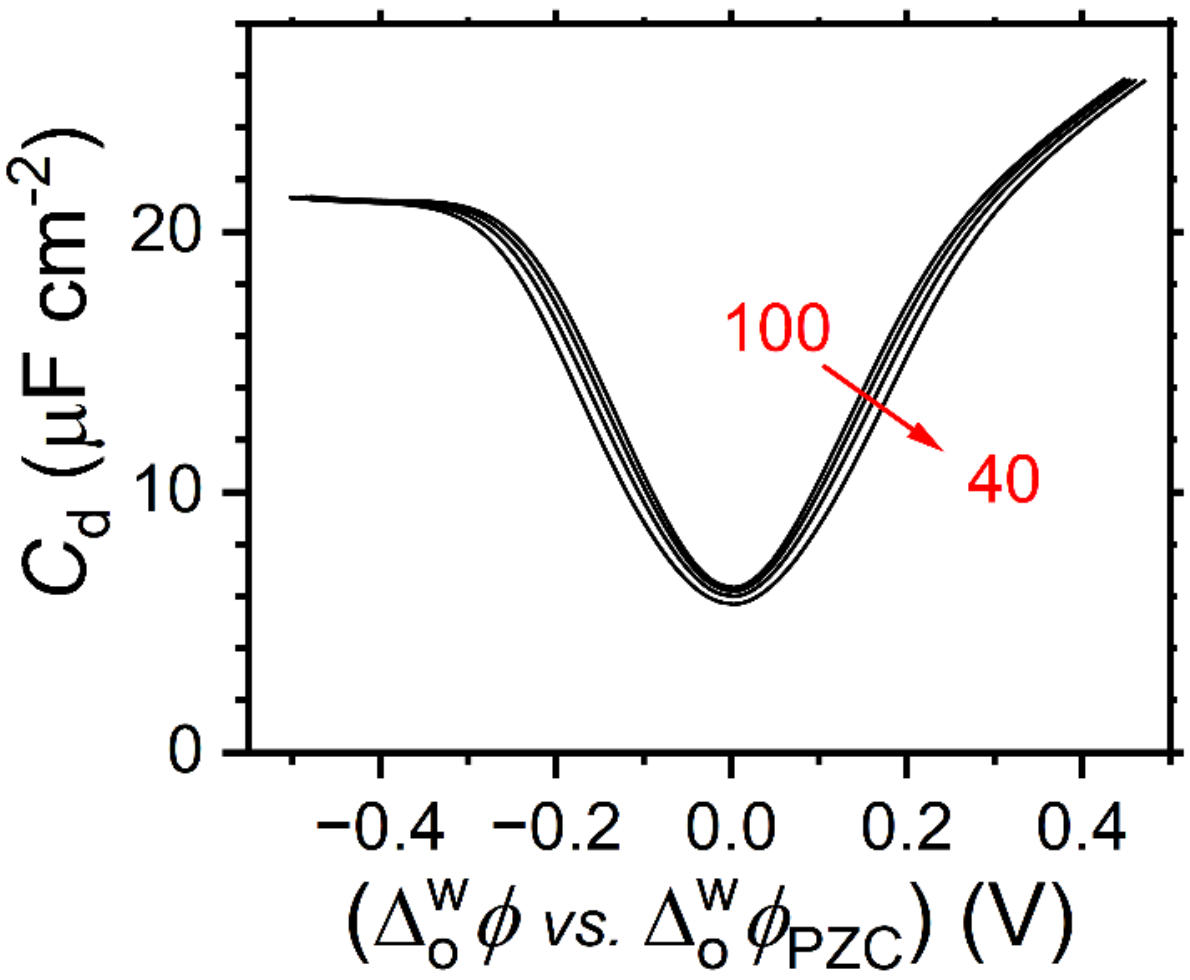

Fig. S8. Effect of the variation of ε_r^w between 40 and 100 in the modelled equivalent capacitance profile, using the C_d^H semiempirical parameters fitted for an ITIES prepared with an aqueous electrolyte solution of 16.0 mM MSA and 5.2 mM BATB in TFT.

When calculating the potential difference vectors across each diffuse layer, the charge symmetry of the electrolyte ions must be considered. For charge-symmetric electrolytes, the equations relating potential difference to interfacial charge density (equations (S1) and (S2)) have an analytical solution (see **Section S1.2**). For example, BATB, used as the organic electrolyte in all cases, is symmetric ($z_{BA^+} = -z_{TB^-} = 1$), so the diffuse layer potential drop can be determined analytically from the charge density using equation (S8). In contrast, Li_2SO_4 is asymmetric ($z_{Li^+} = 1 \neq -z_{SO_4^{2-}} = 2$), meaning that anion and cation charges and concentrations differ. In this case, a numerical method was used to calculate the aqueous diffuse layer potential drop and, in turn, the differential capacitance. Solver was employed to find this potential difference by making zero the squared difference between the initial charge density vector and a second charge density vector calculated using equation (S2). For more clarity on this point, please refer to the tutorial movie available on Zenodo (https://doi.org/10.5281/zenodo.17808336) on how to use the Excel file when modelling the differential capacitance curves. The total error for this calculation step is reported as Σ(% Error σ) in **Tables S8 and S12**. To further investigate how the theoretical model was implemented, the reader can review the Excel files for each cell reported in Zenodo (https://doi.org/10.5281/zenodo.17808336).

The offset in the PZC (ΔPZC) was defined as the difference between the capacitance minimum in the ACV profile and the PZC from the theoretical model, both on the ($\Delta_o^w\phi - \Delta_o^w\phi_{PZC}$) scale. This offset is not a free parameter in the simulation; for instance, it

was not used in modelling LiCl and MSA data, i.e., it was introduced only for asymmetrical-charge electrolytes. In this study, ΔPZC < 0.01 V indicates insufficient resolution of the potential near the capacitance minimum, as is the case for ITIES prepared with Li_2SO_4. The resolution of the potential measurement affects the accuracy of determining the minimum-capacitance point, which serves as the reference for calibrating the potential scale, and consequently can influence the correlation with the model, a problem mitigated by the PZC offset. When ΔPZC is larger, it reflects two possible situations: first, the capacitance at the region near the minimum is wide, the minimum is not clearly identifiable, and therefore the smallest capacitance does not accurately correspond to the PZC, for example, see the data for the ITIES prepared with 1.0 mM Li_2SO_4 and 5.0 mM BATB in TFT (**Fig. 3B(ii)**); second, the model fails to associate the minimum capacitance with the zero interfacial charge density; this is what happens with the ITIES prepared with $CaCl_2$ as the aqueous electrolyte (**Fig. 5**), as discussed in the main text, given more processes are occurring at the liquid|liquid interface.

Table S5. Summary of the fitting variables for the electrochemical cell with **LiCl** as the aqueous electrolyte and **BATB** as the organic electrolyte (see Fig. 3A(i) and A(ii)).

[LiCl] (mM)	[BATB] (mM)	ε_{TFT}	α_1 (F m^{-2})	β_1 (m^2 C^{-1})	γ_1 (F m^{-2})	α_2 (F m^{-2})	β_2 (m^2 C^{-1})	γ_2 (F m^{-2})	ΔPZC (V)
5.0	5.0	25.6	0.939	118.1	0.219	0.294	79.3	0.221	0.0000
10.0	5.0	33.2	1.271	97.3	0.303	1.370	118.0	0.368	0.0000
1000.0	5.0	37.5	0.418	61.5	0.381	1.616	71.9	0.458	0.0000
10.0	1.0	28.7	0.399	57.9	0.170	3.326	392.7	0.269	0.0000
10.0	10.0	31.1	1.057	68.8	0.326	0.879	50.2	0.336	0.0000

Table S6. Summary of the statistical goodness of fit analysis for the electrochemical cell with **LiCl** as the aqueous electrolyte and **BATB** as the organic electrolyte (see Fig. 3A(i) and A(ii)).

[LiCl] (mM)	[BATB] (mM)	χ^2 [a]	SSE [a] (μF^2 cm^{-4})	RMSE [a] (μF cm^{-2})	BIC [a]
5.0	5.0	0.028	1.349	0.138	-49.7
10.0	5.0	0.009	0.815	0.114	-65.7
1000.0	5.0	0.009	1.547	0.181	0.5
10.0	1.0	0.019	0.607	0.108	-55.7
10.0	10.0	0.008	1.052	0.123	-65.1

[a] χ^2 = Non-linear Chi-squared; SSE = Sum of Squared Errors; RMSE = Root Mean Square Error; BIC = Bayesian Information Criterion.

Table S7. Summary of the fitting variables for the electrochemical cell with **Li_2SO_4** as the aqueous electrolyte and **BATB** as the organic electrolyte (see Fig. 3B(i) and B(ii)).

**[Li_2SO_4]** (mM)	**[BATB]** (mM)	ε_{TFT}	**α_1** (F m^{-2})	**β_1** (m^2 C^{-1})	**γ_1** (F m^{-2})	**α_2** (F m^{-2})	**β_2** (m^2 C^{-1})	**γ_2** (F m^{-2})	**ΔPZC** (V)
1.0	5.0	15.1	0.472	130.7	0.182	1.174	224.5	0.229	0.0249
5.0	5.0	22.6	1.004	111.4	0.278	0.693	95.5	0.271	0.0004
500.0	5.0	20.5	0.558	175.0	0.342	0.809	101.0	0.392	0.0005
5.0	1.0	18.1	0.417	374.7	0.185	0.891	378.1	0.173	0.0013
5.0	10.0	21.6	1.039	78.0	0.322	1.071	67.2	0.311	0.0084

Table S8. Summary of the statistical goodness of fit analysis for the electrochemical cell with **Li_2SO_4** as the aqueous electrolyte and **BATB** as the organic electrolyte (see Fig. 3B(i) and B(ii)).

**[Li_2SO_4]** (mM)	**[BATB]** (mM)	χ^2	**SSE** (μF^2 cm^{-4})	**RMSE** (μF cm^{-2})	**BIC**	**Σ (% Error σ)** [a]
1.0	5.0	0.002	0.070	0.037	-163.6	0.0016
5.0	5.0	0.002	0.160	0.054	-132.5	0.0004
500.0	5.0	0.003	0.815	0.123	-40.6	0.0003
5.0	1.0	0.011	0.358	0.086	-67.1	0.0004
5.0	10.0	0.005	0.651	0.106	-62.7	0.0007

[a] Σ (% Error σ) = Total sum of percentage of the absolute differences between the charge density as an independent variable, and the charge density used to calculate the potential drop in the aqueous diffuse layer, the latter was calculated numerically using Solver in Microsoft Excel.

Table S9. Summary of the fitting variables for the electrochemical cell with **MSA** as the aqueous electrolyte and **BATB** as the organic electrolyte (see Fig. 3C(i) and C(ii)).

**[MSA]** (mM)	**[BATB]** (mM)	ε_{TFT}	α_1 ($F\ m^{-2}$)	β_1 ($m^2\ C^{-1}$)	γ_1 ($F\ m^{-2}$)	α_2 ($F\ m^{-2}$)	β_2 ($m^2\ C^{-1}$)	γ_2 ($F\ m^{-2}$)	**ΔPZC** (V)
16.0	5.2	20.6	0.748	56.8	0.375	1.043	50.5	0.279	0.0000
148.0	5.1	18.3	0.281	97.1	0.376	0.606	56.9	0.309	0.0000
1442.0	5.0	23.1	0.368	148.8	0.405	0.511	72.9	0.387	0.0000
16.0	1.3	20.7	1.302	143.6	0.722	1.753	91.5	0.480	0.0000
16.0	10.1	24.0	1.431	67.7	0.406	1.311	44.5	0.265	0.0000

Table S10. Summary of the statistical goodness of fit analysis for the electrochemical cell with **MSA** as the aqueous electrolyte and **BATB** as the organic electrolyte (see Fig. 3C(i) and C(ii)).

**[MSA]** (mM)	**[BATB]** (mM)	χ^2	**SSE** ($\mu F^2\ cm^{-4}$)	**RMSE** ($\mu F\ cm^{-2}$)	**BIC**
16.0	5.2	0.025	1.733	0.108	-205.6
148.0	5.1	0.025	2.608	0.146	-91.0
1442.0	5.0	0.005	0.822	0.094	-143.9
16.0	1.3	0.069	2.554	0.153	-66.7
16.0	10.1	0.015	1.781	0.128	-106.0

Table S11. Summary of the fitting variables for the electrochemical cell with **$CaCl_2$** as the aqueous electrolyte and **BATB** as the organic electrolyte (see Fig. 5B(i) and B(ii)).

**[$CaCl_2$]** (mM)	**[BATB]** (mM)	**ε_{TFT}**	**α_1** ($F\ m^{-2}$)	**β_1** ($m^2\ C^{-1}$)	**γ_1** ($F\ m^{-2}$)	**α_2** ($F\ m^{-2}$)	**β_2** ($m^2\ C^{-1}$)	**γ_2** ($F\ m^{-2}$)	**ΔPZC** (V)
1.1	5.0	**161.3**	0.773	64.9	0.353	0.329	20.7	0.220	0.0306
5.0	5.0	53.0	1.115	49.4	0.355	0.962	44.8	0.277	0.0114
500.7	5.0	16.8	0.479	182.3	0.271	0.397	126.8	0.327	0.0010
1.1	1.0	53.9	8.251	394.4	0.264	8.829	503.3	0.206	0.0053
1.0	10.0	**133.7**	0.746	47.21	0.394	0.537	21.28	0.254	0.0226

Table S12. Summary of the statistical goodness of fit analysis for the electrochemical cell with **$CaCl_2$** as the aqueous electrolyte and **BATB** as the organic electrolyte (see Fig. 5B(i) and B(ii)).

**[$CaCl_2$]** (mM)	**[BATB]** (mM)	χ^2	**SSE** ($\mu F^2\ cm^{-4}$)	**RMSE** ($\mu F\ cm^{-2}$)	**BIC**	**Σ (% Error σ)**
1.1	5.0	**0.051**	4.89	0.193	-19.8	0.0007
5.0	5.0	0.162	19.051	0.358	156.6	0.0008
500.7	5.0	0.003	0.273	0.060	-173.2	0.0034
1.1	1.0	0.170	5.435	0.216	11.3	0.0007
1.0	10.0	**0.472**	38.852	0.533	255.7	0.0011

The red values in **Tables S11** and **S12** indicate cases where, despite achieving a good fit, the ε_{TFT} for ITIES prepared with $CaCl_2$ as the aqueous electrolyte is calculated to be implausibly equal to or even greater than that of water. Such results point to limitations in the measurement method and/or the theoretical model, underscoring the need for careful judgment when interpreting experimental data through a thermodynamic framework.

Section S7. Deconvolution of the differential capacitance contributions for each of the ITIES modelled

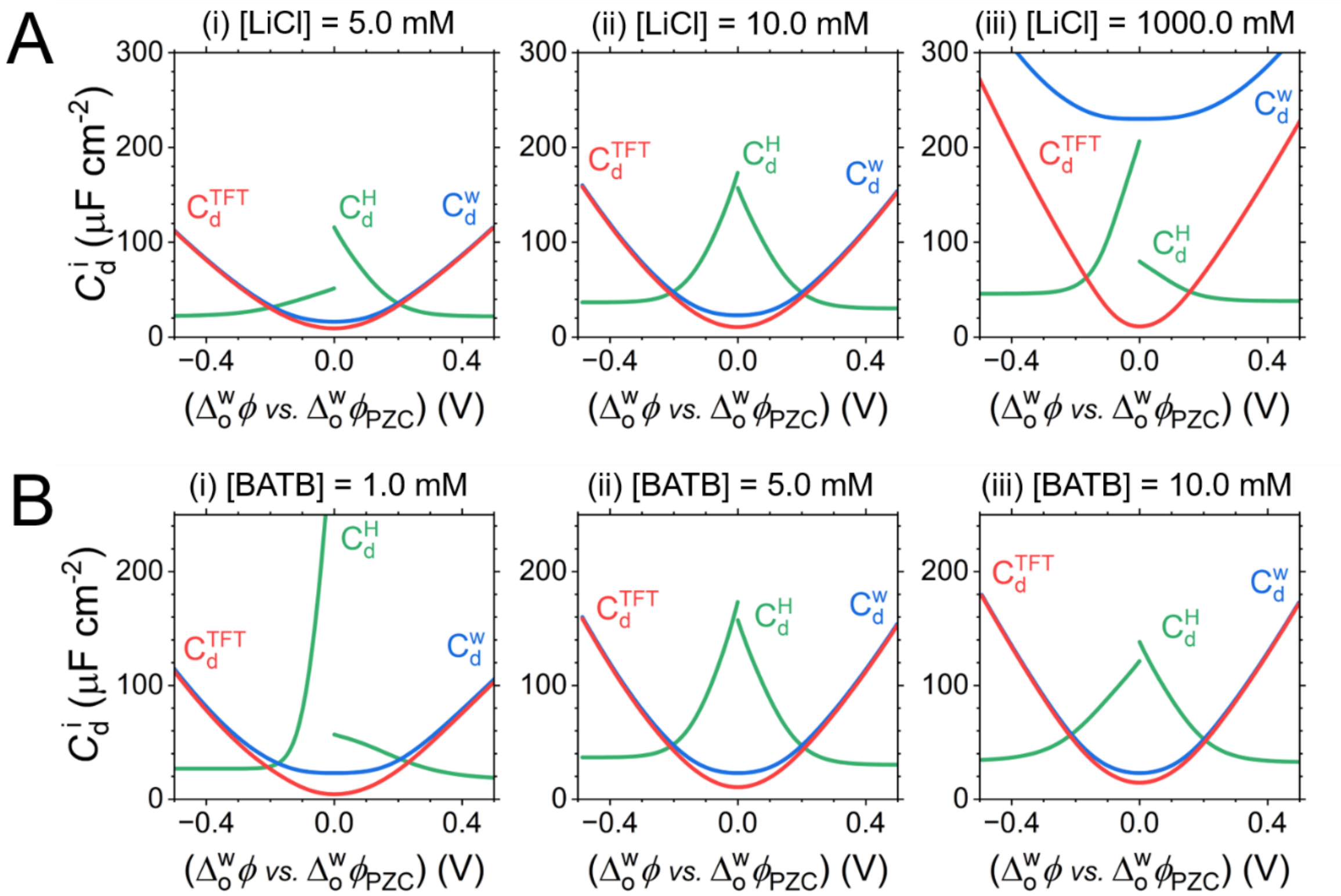

Fig. S9. Contribution of each differential capacitance (C_d^i) component in function of the potential ($\Delta_o^w\phi$ vs. $\Delta_o^w\phi_{PZC}$), for each cell with variable concentrations of **LiCl** as the aqueous electrolyte and **BATB** as the organic electrolyte. The total equivalent capacitance is composed of the capacitance of the organic diffuse layer (C_d^{TFT}, solid red line), the aqueous diffuse layer (C_d^w, solid blue line), and the Helmholtz layer (C_d^H, solid green line). The fixed concentrations employed were: (**A**) [BATB] = 5.0 mM; (**B**) [LiCl] = 5.0 mM. The variable concentrations are indicated in each panel.

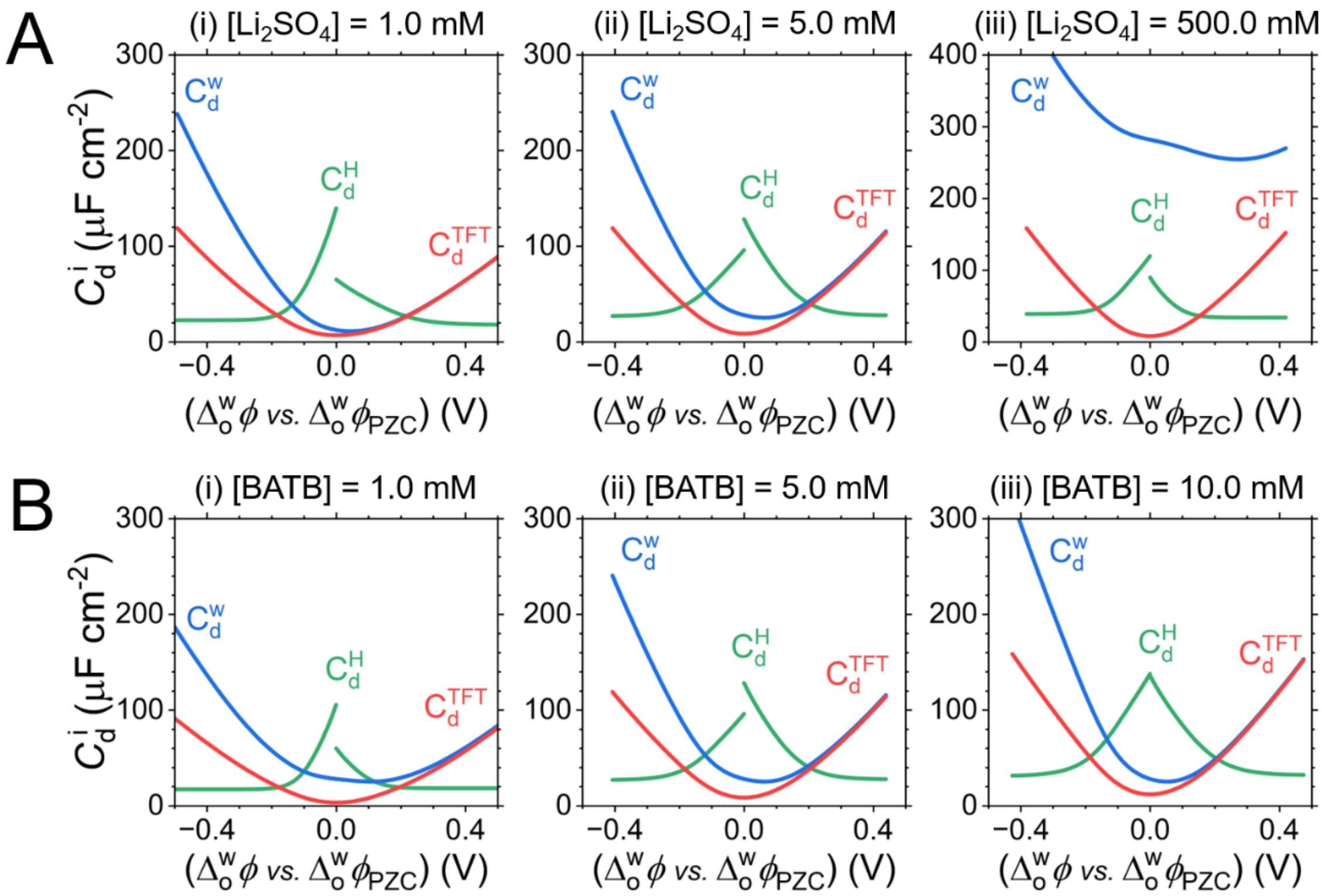

Fig. S10. Contribution of each differential capacitance (C_d^i) component in function of the potential ($\Delta_\mathrm{o}^\mathrm{w}\phi$ vs. $\Delta_\mathrm{o}^\mathrm{w}\phi_\mathrm{PZC}$), for each cell with variable concentrations of **Li_2SO_4** as the aqueous electrolyte and **BATB** as the organic electrolyte. The total equivalent capacitance is composed of the capacitance of the organic diffuse layer ($C_\mathrm{d}^\mathrm{TFT}$, solid red line), the aqueous diffuse layer (C_d^w, solid blue line), and the Helmholtz layer (C_d^H, solid green line). The fixed concentrations employed were: (**A**) [BATB] = 5.0 mM; (**B**) [Li_2SO_4] = 5.0 mM. The variable concentrations are indicated in each panel.

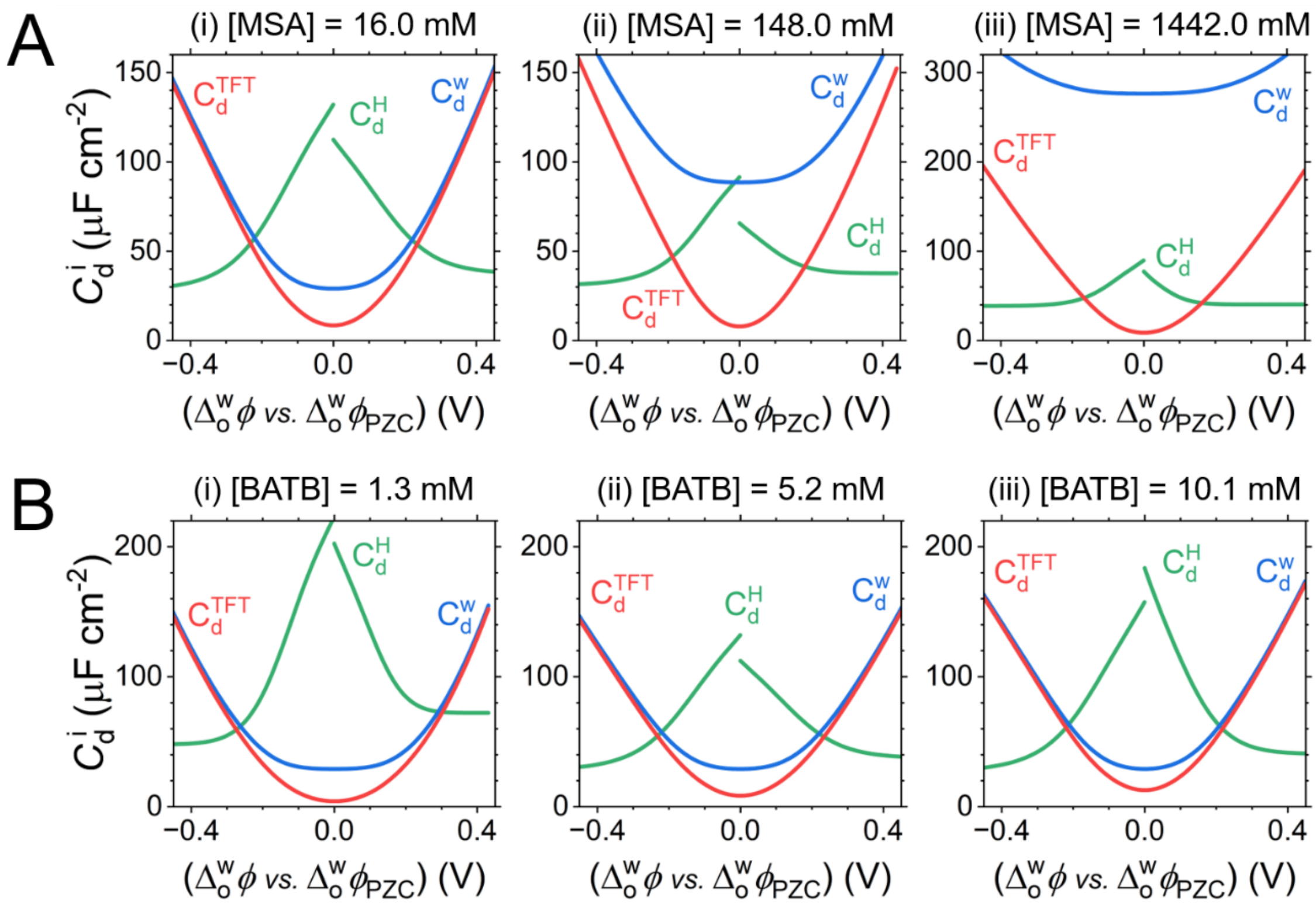

Fig. S11. Contribution of each differential capacitance ($C_{\mathrm{d}}^{\mathrm{i}}$) component in function of the potential ($\Delta_{\mathrm{o}}^{\mathrm{w}}\phi$ vs. $\Delta_{\mathrm{o}}^{\mathrm{w}}\phi_{\mathrm{PZC}}$), for each cell with variable concentrations of **MSA** as the aqueous electrolyte and **BATB** as the organic electrolyte. The total equivalent capacitance is composed of the capacitance of the organic diffuse layer ($C_{\mathrm{d}}^{\mathrm{TFT}}$, solid red line), the aqueous diffuse layer ($C_{\mathrm{d}}^{\mathrm{w}}$, solid blue line), and the Helmholtz layer ($C_{\mathrm{d}}^{\mathrm{H}}$, solid green line). The fixed concentrations employed were: (**A**) [BATB] = 5.0 mM; (**B**) [MSA] = 16.0 mM. The variable concentrations are indicated in each panel.

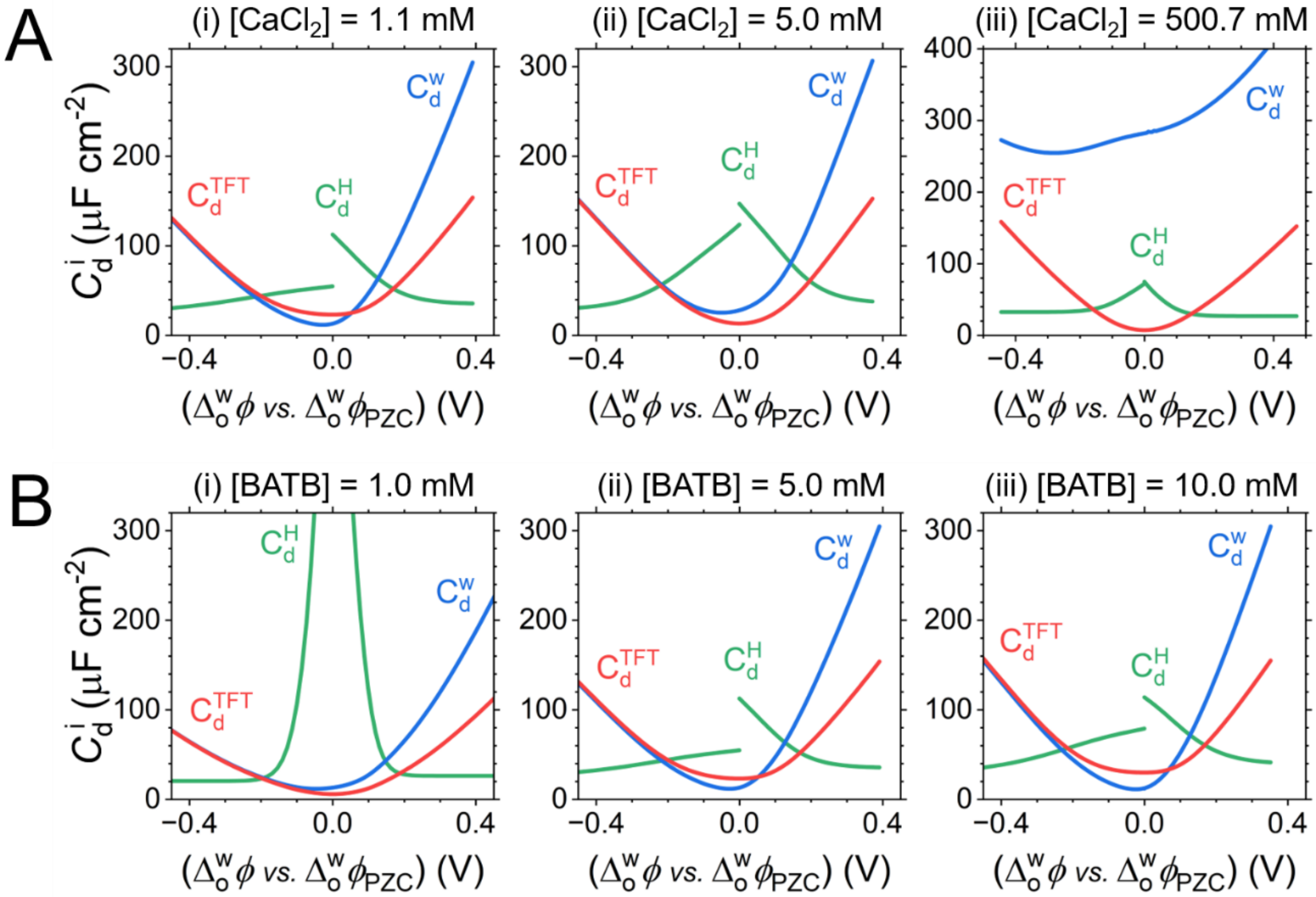

Fig. S12. Contribution of each differential capacitance (C_d^i) component in function of the potential ($\Delta_o^w\phi\ vs.\Delta_o^w\phi_{PZC}$), for each cell with variable concentrations of **$CaCl_2$** as the aqueous electrolyte and **BATB** as the organic electrolyte. The total equivalent capacitance is composed of the capacitance of the organic diffuse layer (C_d^{TFT}, solid red line), the aqueous diffuse layer (C_d^w, solid blue line), and the Helmholtz layer (C_d^H, solid green line). The fixed concentrations employed were: (**A**) [BATB] = 5.0 mM; (**B**) [$CaCl_2$] = 1.1 mM. The variable concentrations are indicated in each panel.

Supporting references

1 R. A. Lehane, A. Gamero-Quijano, J. A. Manzanares and M. D. Scanlon, *J. Am. Chem. Soc.*, 2024, **146**, 28941–28951.

2 E. Smirnov, P. Peljo, M. D. Scanlon and H. H. Girault, *Electrochim. Acta*, 2016, **197**, 362–373.

3 A. Gamero-Quijano, G. Herzog and M. D. Scanlon, *Electrochem. Commun.*, 2019, **109**, 106600.

4 A. K. Bain and P. Chand, in *Pyroelectric Materials: Physics and Applications*, Wiley, 1st edn., 2022.

5 M. F. Suárez-Herrera and M. D. Scanlon, *Electrochim. Acta*, 2019, **328**, 135110.

6 A. Trojánek, V. Mareček and Z. Samec, *Electrochim. Acta*, 2015, **179**, 3–8.

7 M. F. Suárez-Herrera and M. D. Scanlon, *Anal. Chem.*, 2020, **92**, 10521–10530.

8 Z. Samec, A. Lhotský, H. Jänchenová and V. Mareček, *J. Electroanal. Chem.*, 2000, **483**, 47–56.

9 Z. Samec, V. Mareček and D. Homolka, *Faraday Discuss. Chem. Soc.*, 1984, **77**, 197–208.

10 Y. Marcus, *J. Solution Chem.*, 2013, **42**, 2354–2363.
